# Unlocking Potentially Therapeutic Phytochemicals in Capadulla (*Doliocarpus dentatus*) from Guyana Using Untargeted Mass Spectrometry-Based Metabolomics

**DOI:** 10.3390/metabo13101050

**Published:** 2023-10-03

**Authors:** Ewart Smith, Ainsely Lewis, Suresh S. Narine, R. J. Neil Emery

**Affiliations:** 1 Environmental and Life Sciences Graduate Program, Trent University, Peterborough, ON K9J 0G2, Canada; 2 Department of Biology, Trent University, Peterborough, ON K9J 0G2, Canada; 3 Trent Centre for Biomaterials Research, Trent University, Peterborough, ON K9J 0G2, Canada; 4 Departments of Physics & Astronomy and Chemistry, Trent University, Peterborough, ON K9J 0G2, Canada

**Keywords:** Capadulla, erectile dysfunction, mass spectrometry, metabolomics, polyphenols, antioxidants

## Abstract

*Doliocarpus dentatus* is thought to have a wide variety of therapeutic phytochemicals that allegedly improve libido and cure impotence. Although a few biomarkers have been identified with potential antinociceptive and cytotoxic properties, an untargeted mass spectrometry-based metabolomics approach has never been undertaken to identify therapeutic biofingerprints for conditions, such as erectile dysfunction, in men. This study executes a preliminary phytochemical screening of the woody vine of two ecotypes of *D. dentatus* with renowned differences in therapeutic potential for erectile dysfunction. Liquid chromatography–mass spectrometry-based metabolomics was used to screen for flavonoids, terpenoids, and other chemical classes found to contrast between red and white ecotypes. Among the metabolite chemodiversity found in the ecotype screens, using a combination of GNPS, MS-DIAL, and SIRIUS, approximately 847 compounds were annotated at levels 2 to 4, with the majority of compounds falling under lipid and lipid-like molecules, benzenoids and phenylpropanoids, and polyketides, indicative of the contributions of the flavonoid, shikimic acid, and terpenoid biosynthesis pathways. Despite the extensive annotation, we report on 138 tentative compound identifications of potentially therapeutic compounds, with 55 selected compounds at a level-2 annotation, and 22 statistically significant therapeutic biomarkers, the majority of which were polyphenols. Epicatechin methyl gallate, catechin gallate, and proanthocyanidin A2 had the greatest significant differences and were also relatively abundant among the red and white ecotypes. These putatively identified compounds reportedly act as antioxidants, neutralizing damaging free radicals, and lowering cell oxidative stress, thus aiding in potentially preventing cellular damage and promoting overall well-being, especially for treating erectile dysfunction (ED).

## 1. Introduction

Doliocarpus dentatus, popularly known as Guyanese Capadulla, is an important medical woody stem belonging to the Dilleniaceae family [1,2]. The *D. dentatus* species is commonly found in tropical rainforests in Central and South Americas, from Mexico to South Brazil, and the Amazon region has the highest representation of the species [3]. In Guyana, the woody vine of *D. dentatus* grows in mixed to evergreen lowland forests [4,5,6,7]. The Capadulla woody vine is regarded locally as a powerful aphrodisiac [8,9]. The stem of the *D. dentatus* is used to make tea or drinks, which purportedly raises libido, cures impotence, and improves sexual desire in men [9].

In Guyana, the traditional use of *D. dentatus* has involved treating specific symptoms of diabetes, Leishmanial ulcers, and erectile dysfunction (ED), and supporting overall well-being [10,11]. 

The differences between *D. dentatus* red and white ecotypes are recognized by their wood color instead of their botanical features, such as leaves and fertile organs [12]. Two ecotypes of *D. dentatus* (i.e., white and red) are used similarly as an aphrodisiac without reported noticeable differences in their efficacy by Guyanese locals. Informants have also indicated that the *D. dentatus* red species is considered a better aphrodisiac than the Capadulla white ecotype, and that “Capadulla red is considered a male species consumed by males”. At the same time, “Capadulla white is regarded as the female species but is consumed when Capadulla red is in limited supply”. 

In earlier studies on *D. dentatus*, using mass spectrometry, total phenolic (204.04 mg/g), flavonoid (89.17 mg/g), and tannins (12.05 mg/g) contents, and sitosterol-3-O-D-glucopyranoside, kaempferol 3-O-L-aminopyranoside, betulinic acid, and betulin were discovered in the ethanolic extract of *D. dentatus* leaves, which evaluated for widespread use against pain [13]. An additional phytochemical analysis confirmed the presence of butyric acid, steroids, lactones, anthracensides, betulinic acid, tannins, flavones, and phenolic acids in this plant [14]. 

*D. dentatus* leaves’ ethanolic extract was evaluated for widespread use against pain in animal models and its toxicogenicity [15]. The results indicated that an ethnopharmacological dose of the aqueous extract from the leaves of *D. dentatus* decreased the pain response, indicating analgesic and anti-inflammatory actions. Furthermore, toxicogenic assays determined that the aqueous extract of *D. dentatus* did not have genotoxic potential and did not modify splenic phagocytosis [3,15]. Another study suggested that the extract from the leaves of *Capadulla* had antibacterial and anti-inflammatory properties, demonstrating that the leaves had phytochemical compounds that could treat various illnesses [13,16]. The extract from the leaves of *D. dentatus* is safe to use during the gestational period. It does not cause DNA damage and is not teratogenic [16]. 

In vitro studies of the biological activity of *D. dentatus* have shown that it is an antimicrobial against strains of *Escherichia coli*, *Klebsiella pneumoniae*, and *Staphylococcus aureus*; anti-inflammatory; and cytotoxic in leukemic cells of the K562 lineage [13]. It is effective against amastigotes of *Leishmania amazonenses* [17,18] and does not cause genomic or chromosomal damage, implying that it is safe for use [14,15,16,19]. 

Although many phytochemicals have been discovered in *D. dentatus leaves,* the woody vine of *Doliocarpus dentatus* has not been investigated using mass spectrometry-based metabolomics and other bioinformatics techniques. The objective of the current study is to determine the phytochemical profile of *D. dentatus* ecotypes utilizing untargeted and semi-targeted plant metabolomics using liquid chromatography (LC) coupled with electrospray ionization mass spectrometry (ESI-MS/MS). Liquid chromatography–electrospray ionization-–mass spectrometry (LC-ESI-MS) is a sensitive technology for determining compound molecular masses. ESI-tandem mass spectrometry (MS/MS) can be utilized to obtain additional structural information on compound fragmentation patterns [20,21]. LC-MS-based metabolomics methods are commonly used to profile complex biological extracts, such as those from plants [22,23], with non-targeted plant metabolomics being of particular interest, owing to the large plant kingdom’s metabolite variety [24,25]. 

The goal of this work is to perform a preliminary investigation of the phytochemical profiles of *D. dentatus* that will provide additional information on the potential use of the compounds, compare compounds in both ecotypes, and identify any unique or beneficial compounds present in *D. dentatus* using mass spectrometry-based metabolomics. This information can be used to better understand the physiology and ecology of this species and potentially inform conservation efforts. It may also affect human health, as *D. dentatus* is a known source of bioactive compounds. Additionally, understanding the metabolic pathways of this plant can eventually help target new therapies for erectile dysfunction in men. 

## 2. Materials and Methods

### 2.1. Chemicals Used for Metabolite Extraction and Liquid Chromatography–Mass Spectrometry

HPLC-grade solvents, such as acetonitrile and methanol, were purchased from Fisher Scientific (Hampton, NH, USA). Water used for metabolite extraction and the mobile phase in liquid chromatography–mass spectrometry (LC-MS) was previously purified using a Milli-Q system (Millipore, Burlington, MA, USA). Benzyladenine and abscisic acid-labeled standards were acquired from OlChemim Ltd., Olomouc, Czech Republic. 

### 2.2. Study Site and Plots–Eagle Mountain Forest Potaro–Siparuni, Guyana

The Eagle Mountain Forest is about 200 km south–southwest of Georgetown, Guyana’s capital city (21N0261909-0578704). The Eagle Forest, located in an isolated area, is characterized by steep sandstone ridges ranging in height from 100–724.8 m above sea level. Annual rainfall is very high (3500–4000 mm), with noticeable peaks in May and June. The study area is ecologically significant, supporting a high diversity of flora and fauna. It is dominated by Leguminosae/Fabaceae subfamilies (Mimosaceae, Caesalpiniaceae, and Papilionaceae) [26,27]. *D. dentatus* liana was collected randomly after an inventory of all *D. dentatus* lianas had been created and their position recorded. The measurement point for all *D. dentatus* liana was 3–5 cm representing seedlings, and poles, 20 cm and longer, representing mature individuals in the diameter range, known to capture the demography for seedling poles and mature individuals [28]. In July 2022, we collected a total of four biological samples, all of which were chosen at random from the *D. dentatus* liana population present at the Eagle Mountain study location. Collections included red and white *D. dentatus*, utilizing diameter classes (50–200 mm). 

For every individual *D. dentatus* liana selected, a 20 mm disc was carefully extracted from various points along the woody vine, encompassing the ground level, an intermediate position, and an emergent point. The processed samples of the same ecotypes with similar diameters formed a composite sample. Following the extraction, these samples were promptly placed in dry ice to maintain their metabolic integrity during transit. Subsequently, they were transported to the University of Guyana (Greater Georgetown, Guyana). Here, the samples underwent further processing to prepare them for an in-depth analysis. Finally, the samples were sent to Trent University (Peterborough, Canada), where an advanced metabolomic analysis was conducted.

### 2.3. Biological Sample Preparation and Metabolite Extraction

Metabolite extraction was performed as previously published [29]. Thirty mg of *D. dentatus* dried tissues were pre-weighed before the extraction. Tissues in 2 mL Eppendorf tubes with 2 zirconium grinding beads (Comeau Technique Ltd., Vandreuil-Dorion, QC, Canada) were loaded into a Retsch MM300 ball mill (Haan, Germany) where they were shaken for 5 minutes at 25 Hz. The powder was suspended in 1 mL of ice-cooled 80% MeOH/20% double-distilled water *v*/*v* and sonicated for 15 min. The tubes were centrifuged (Sorvall ST 16 centrifuge; ThermoScientific, Waltham, MA, USA) at 10,000 rpm for 10 min. A total of 700 μL of sample supernatant was transferred to 0.2 μM PVDF centrifuge filters with 2 mL receiver tubes and spun down at 10,000 rpm for 5 min. Filtered extracts were dried at ambient temperature in the speed vacuum centrifuge (ThermoScientific Savant SpeedVac^TM^, Rockford, IL, USA). Residues were redissolved in 400 μL 50% MeOH/water and spun down again at 10,000 rpm. A total of 300 μL of supernatant was transferred to a 350 μL autosampler insert inside a 2 mL glass vial with a septa cap. A total of 10 μL (10 ng) of benzyladenine and 27 μL (59 ng) of abscisic acid-labeled standards (OlChemim Ltd., Olomouc, Czech Republic) were added to each vial to monitor the retention time deviation across samples for positive and negative ionization modes, respectively.

### 2.4. Data Acquisition via Liquid Chromatography–Mass Spectrometry (LC-MS)

Extracts from both *D. dentatus* ecotypes (i.e., red and white) were analyzed by LC-MS [29] with minor modifications. For an untargeted metabolomics analysis, the data were acquired using a Thermo Q-Exactive Orbitrap mass spectrometer (Thermo Scientific, San Jose, CA, USA) coupled to a Thermo Dionex Ultimate 3000 Liquid Chromatograph System (Thermo Scientific, San Jose, CA, USA). All samples were injected for analysis in a mixed mode (positive and negative electrospray ionization (ESI) modes) with an initial mass range of *m*/*z* 80−600 at a resolution of 140,000 at *m*/*z* 200 full width at half minimum (FWHM), with an automatic gain control (AGC) target of 3 × 10^6^, and maximum injection time (IT) of 524 ms. The following conditions were used for the heated electrospray ionization (HESI) probe: capillary temperature, 250 °C; sheath gas, 30 arbitrary units; auxiliary gas, 8 units; probe heater temperature, 450 °C; S-Lens rf level, 60%; and capillary voltage, 3.9 kV.

Quality controls (QCs) composed of equal volumes of sample replicates, were used for data-dependent tandem mass spectrometry (ddms^2^). QCs were created with a mixture of all replicates from both ecotypes and also from each ecotype. Fragmentation using ddms^2^ was performed at a resolution of 17,500 with an AGC target of 5 × 10^5^ within a narrower mass range of 100 to 600 *m*/*z*. The fragmentation was triggered at a loop count of 10 (top 10 most intense peaks per scan), with a precursor isolation window of 1 *m*/*z* at a normalized collision energy (NCE) of 35 eV. The maximum IT was 64 ms. 

The chromatographic separation was accomplished with an HGP-3400RS dual pump and WPS-3000 autosampler equipped with a Kinetex C_18_ column (2.1 i.d × 50 mm, 2.6 μm particle size, Phenomenex, Torrace, CA, USA) operated at an approximate room temperature of 22 °C. The instrument control was achieved with Chromeleon Chromatography Data System software version 6.8 (ThermoScientific; Ottawa, ON, Canada). For the separation, component A comprised ddH_2_0 with 0.1% formic acid, and component B comprised acetonitrile (CH_3_CN) with 0.1% formic acid, at a flow rate of 0.3 mL/min. Mobile phase B was held at 0% for 30 s, before increasing it to 100% over 3 min. Solvent B was then held at 100% for 2 min before returning to 0% over 4 min for column re-equilibration. The total runtime was 10 min, with a sample injection volume of 25 μL. 

### 2.5. Data Processing of Untargeted Mass Spectrometry Data 

The MS Convert utility (Version 3.0) of the open-source ProteoWizard program was used to convert raw mass spectrometric data into an mzXML format [30]. As the LC-MS acquisition occurred in the mixed ionization mode, negative and position ions were extracted separately by using the default for peak picking and the threshold peak filter with absolute intensity using the most intense ions (0.001 and above). Peak peaking, retention time correction, and peak grouping were all conducted using the default parameters of XCMS Online [31], which is included in the Supplementary Materials. The pairwise option in XCMS Online was used with the white ecotype as the control [32]. After peak peaking, retention time correction, and peak grouping, the resulting peak intensity matrix was subjected to a statistical analysis using MetaboAnalyst 5.0 [32]. The data were normalized by sum, and then log-transformed before a principal component analysis (PCA) to visualize the overall clustering pattern of the samples. Additionally, a partial least squares discriminant analysis (PLS-DA) was performed to identify the metabolites that contributed most to the separation between sample groups. The significance of differential metabolites was determined using the *t*-test and fold-change analysis.

*D. dentatus* red and white putative MS^1^ *m*/*z* features were compared to databases of physiologically active substances in the literature and with bioinformatics servers used in untargeted metabolomics, such as KnapSack [33], KEGG [34], the Plant Metabolic Network using the PlantCyc database (https://plantcyc.org, accessed on 10 June 2023) [35], MetaboQuest [36], SciFinder [37], and PubChem [38]. In the processing module of XCalibur 4.1 (ThermoScientific, Waltham, MA, USA), we inputted the putative identities of key therapeutic compound families (i.e., flavonoids, terpenoids, and alkaloids). They extracted the exact masses of protonated or deprotonated compounds.

The putative identities of the key therapeutic compound families (i.e., flavonoids, terpenoids, and alkaloids) generated were inputted in the processing module in XCalibur 4.1 (ThermoScientific, Waltham, MA, USA) where the exact masses of protonated or deprotonated compounds were extracted. This semi-targeted approach was performed to determine peak quality and to monitor the peaks within the samples for therapeutic compounds falling under polyphenolics, terpenoids, and alkaloids. The relative quantification of peak areas was performed to determine up-or-downregulated compounds, by the ratio of the averages of [Capadulla Red]/[Capadulla White]. If the ratio (fold change; FC) > 1, the samples were upregulated. The converse was downregulated if the ratio < 1 (Supplemental Figure S2). 

**Figure 1:**
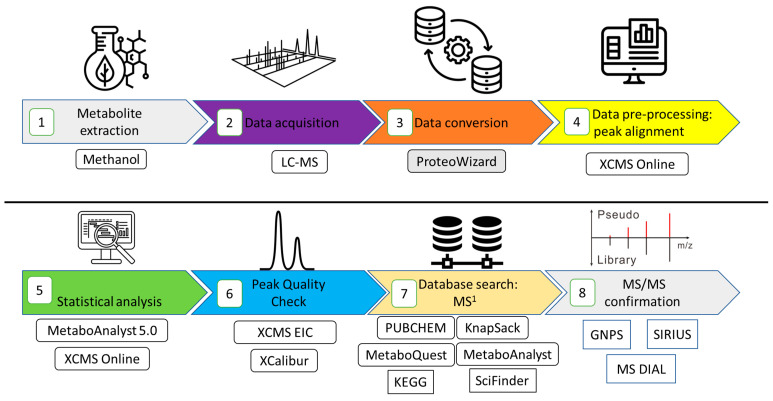
Metabolomics workflow used for extracting, acquiring, and analyzing compounds present in *D. dentatus* ecotypes. Icons used are sourced from The Noun Project.

Metabolite Autoplotter was used to generate images for tentative metabolites with corresponding peak areas [39] The workflow for data processing can be seen in Figure 1 for MS^1^ data.

### 2.6. Statistical Analysis of Metabolomics Datasets

Untargeted metabolomic analyses generate complex multivariate datasets with thousands of tentative metabolite features and their corresponding intensities. Various dimensionality reduction procedures are employed to simplify the data and understand the relationships among the samples based on these intensities. Metabolomic data analysis is characterized by high dimensionality, where the number of measured metabolite features exceeds the number of samples and collinearity among the features. These factors challenge traditional linear regression methods, necessitating statistical procedures capable of handling collinearity [40].

Before any statistical analysis, preprocessing steps were applied to normalize the data to facilitate further analyses. This involved sum normalization, log_2_ transformation, and Pareto scaling to ensure the appropriate scaling of the obtained data using the MetaboAnalyst 5.0 platform [41] (www.metaboanalyst.ca, accessed on 10 June 2023). By normalizing the data through sum normalization, applying a log_2_ transformation, and performing Pareto scaling, the dataset was prepared for a subsequent statistical analysis. These preprocessing steps helped to ensure that the data were appropriately scaled and standardized, allowing for meaningful comparisons and accurate statistical inference. We used ANOVA and the volcano plot to find metabolites strongly affected by the experimental factor, which was the difference between the red and white ecotypes.

The XCMS Online software tool was utilized to annotate the LC-MS dataset, which was subsequently analyzed through the MetaboAnalyst 5.0 platform. The methodology was employed to identify the differential metabolites involved using the standard volcano plot, which integrated the *t*-test and fold-change techniques. The graph displays the logarithmic transformation of the fold-change values on the X-axis and the negative logarithmic transformation of the *p*-values obtained from the *t*-test on the Y-axis [42].

Furthermore, this analysis seeks to uncover potential *m*/*z* biofingerprints that are elevated or downregulated in *D. dentatus* red and white ecotypes that may have therapeutic potential for erectile dysfunction. These statistical approaches, combined with the visual representation provided by the volcano plot, principal component analysis (PCA), partial least-squares discriminant analysis (PLS-DA), and hierarchical cluster analysis (HCA), offer valuable insights into the metabolomic variations between the *D. dentatus* red and white ecotypes. 

Principal component analysis and partial least-squares discriminant analysis are commonly employed to reduce the dimensionality of the data and identify differences between the groups. PCA aims to replace correlated variables with uncorrelated variables, known as principal components (PCs), which capture most of the variability in the original dataset. This allows for initial biological conclusions about the samples, which can be further verified using PLS-DA or orthogonal projections to latent structure DAs (OPLS-DAs). Despite having fewer samples than variables (metabolite features and intensities), multivariate statistical methods, such as PCA [43,44], PLS-DA [45], and HCA [46], are appropriate for analyzing metabolomics datasets [47,48,49,50,51,52,53].

In our study, we performed a PCA to examine the relationships among samples based on the relative intensities of all tentative metabolite features. PCA groups formed into clusters based on the overall similarity or metabolite profile differences. Samples with similar profiles were grouped together on the PCA plot. PCA determines these relationships solely based on the metabolite features without considering sample descriptions, such as species or treatment. Following determining the sample relationships through PCA, we identified the top 25 significantly different features (*p* ≤ 0.05) using PLS-DA or OPLS-DA. These features allowed for separating the samples into distinct groups, indicating significant differences in their accumulation behavior among ecotypes and treatments. 

Additionally, HCA was conducted to identify the top 25 statistically significant tentative metabolite features between the ecotypes in both positive and negative modes. Pearson’s correlation was used to assess the relatedness of samples based on these 25 features (Figures 6 and 7). The results of the HCA are visualized as a heatmap, with the samples and features displayed on the X- and Y-axes, respectively, and relative metabolite intensities indicated by a color scale. The relationship among the samples is depicted using a color-coded dendrogram atop the HCA plot. Similar to PLS-DA, HCA identifies metabolite features that separate the samples into distinct clusters aligned with sample descriptions. Ward’s clustering algorithm and Pearson’s correlation were employed to construct the dendrograms, with the Euclidean distance indicated on the X-axis. All statistical analyses were performed using the MetaboAnalyst 5.0 platform [41] (Pang, Chong et al., 2021).

### 2.7. Tandem Mass Spectrometry-Data Analysis for Metabolite Annotation 

**Figure 2:**
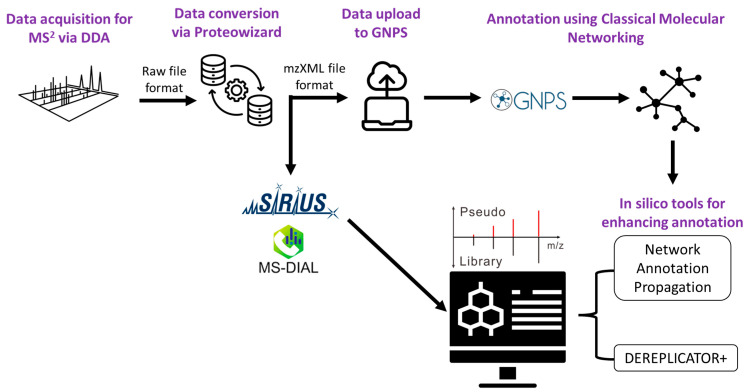
Processing of tandem mass spectrometry data using a combination of bioinformatics tools for level 2–3 annotations.

Using diverse bioinformatic tools can aid in metabolite annotation. Global Nature Production Social Molecular Networking (GNPS), SIRIUS, and MS-DIAL were therefore used to query the fragments for putative identification [3,54]. The metabolite annotation levels using these tools were level 2 for MS-DIAL, levels 2 and 3 for GNPS, and level 3 using the in silico fragmentation tool SIRIUS, and were assigned these levels as recommended by the Metabolomics Standards Initiative [55]. The workflow for the data analysis can be seen in Figure 2.

#### 2.7.1. Metabolite Annotation Using MS-DIAL 

Tandem mass spectrometry raw data were processed with MS-DIAL 5.1 [56]. Automatic feature detection was performed between retention times of 0 and 10 min for mass signal extractions in positive and negative ionization modes. MS^1^ and MS^2^ tolerance values were set to 0.01 and 0.025 Da, respectively, in the profile mode. Minimum feature height, mass slice width, and the sigma window value were all set to the default of 1000 (Aus: arbitrary units), 0.1 Da, and 0.5, respectively [57]. Alignments for samples were performed at a 0.015 Da mass tolerance and 0.05 min for the retention time tolerance for MS^1^. Database matches were performed with the parameters of MS^1^ and MS^2^ at 0.01 and 0.05 Da, respectively (instructions as previously published [58]. Databases for both positive and negative ionization modes were downloaded and chosen for level 2 from the MS-DIAL website (<http://prime.psc.riken.jp/compms/msdial/main.html> accessed on 1 March 2023).

#### 2.7.2. Metabolite Annotation Using GNPS via Classical Molecular Networking 

Raw data from the quality control samples from each ecotype were converted to mzXML via MSConvert option in Proteowizard and submitted to GNPS. A molecular network for both negative and positive ionization data was created using the online workflow (https://ccms-ucsd.github.io/GNPSDocumentation/, accessed on 14 September 2023) on the GNPS website (http://gnps.ucsd.edu, accessed on 14 September 2023). The data were filtered by removing all MS/MS fragment ions within +/− 17 Da of the precursor *m*/*z*. MS/MS spectra were window filtered by choosing only the top 6 fragment ions in the +/− 50 Da window throughout the spectrum. The precursor ion mass tolerance was set to 0.02 Da and a MS/MS fragment ion tolerance of 0.02 Da was adopted. A network was then created where the edges were filtered to have a cosine score above 0.6 and more than 3 matched peaks. Furthermore, edges between the two nodes were kept in the network if and only if each of the nodes appeared in each other’s respective top 10 most similar nodes. Finally, the maximum size of a molecular family was set to 100, and the lowest scoring edges were removed from molecular families until the molecular family size was below this threshold. The spectra in the network were then searched against GNPS’s spectral libraries. The library spectra were filtered in the same manner as the input data. All matches kept between the network and library spectra were required to have a score above 0.6 and at least 3 matched peaks [59].

The workflow and results of classical molecular networking can be found in the following GNPS repositories for positive and negative ionization data (Task IDs = e06a20c58fa04355ba3630912077b3ea and 8ceaa322aac44c62a027d131b33dc4ce).

Dereplicator+ [60] and Network Annotation Propagation (NAP) [61] were used in conjunction for enhanced annotations. Although MolNetEnhancer [62] was a workflow used for combining the results of CMN, Dereplicator+, and NAP, this function did not work for annotating our results for the visualization of compound classes via Cytoscape [63]. However, overall visualization was performed in Cytoscape 3.10.0 after merging the networks of both negative and positive ionization modes [64] (included in Supplementary Materials). 

Workflows for Dereplicator+ and NAP can be found at (Task ID for NAP: 62fec77af3ac485aa4ae473dec025e3e for the positive ionization mode and 7e718792512d4acbaeafe17952ae4f28 for the negative ionization mode. For Dereplicator+, the task IDs were 5ed74a5b3b3e47eb904ec329b9fc73cd for the positive ionization mode and 95247d7f7dbf4297963f7f451ac88ff3 for the negative ionization mode. For the merged polarity network, the task ID was 8f393dfcc9fc492c88d6d8cc6979d1c4.

#### 2.7.3. Metabolite Annotation Using GNPS via Feature-Based Molecular Networking

The mzMine 2.5.3 analysis pipeline [65,66] was used to look for MS^1^ features corresponding to MS^2^ fragments. The data acquired from the QC samples from each ecotype analyzed using ddMS^2^ were converted to the mzXML format using the MSConvert option in Proteowizard, as mentioned previously. The peaks were aligned using the previously published parameters with modifications involving mass detection, chromatogram building, smoothing deconvolution, deisotoping, alignment, and gap filling [67]. All the data (for MS^1^ and MS^2^) were used and stored in.mgf format with the corresponding.csv file. 

A molecular network was created with the Feature-Based Molecular Networking (FBMN) workflow [68] on GNPS (https://gnps.ucsd.edu, accessed on 14 September 2023) [59]. The results from mzMine 2.5.3 were exported to GNPS for the FBMN analysis. FBMN was used to resolve any isomers and also to check for any other annotations due to cleaning up the data during mzMine preprocessing. The data were filtered by removing all MS/MS fragment ions within +/− 17 Da of the precursor *m*/*z*. MS/MS spectra were window filtered by choosing only the top 6 fragment ions in the +/− 50 Da window throughout the spectrum. The precursor ion mass tolerance was set to 0.02 Da and the MS/MS fragment ion tolerance to 0.02 Da. A molecular network was then created where the edges were filtered to have a cosine score above 0.6 and more than 3 matched peaks. Furthermore, the edges between two nodes were kept in the network if and only if each of the nodes appeared in each other’s respective top 10 most similar nodes. Finally, the maximum size of a molecular family was set to 100, and the lowest scoring edges were removed from molecular families until the molecular family size was below this threshold. The spectra in the network were then searched against GNPS spectral libraries [59,69]. The library spectra were filtered in the same manner as the input data. All matches kept between network and library spectra were required to have a score above 0.6 and at least 3 matched peaks. The DEREPLICATOR was used to annotate the MS/MS spectra [60]. The molecular networks were visualized using Cytoscape software [63].

The workflow and results of FBMN networking can be found in the following GNPS repositories for positive and negative ionization data (Task IDs = 39963758986b4f09b0b992d130489534 and 031003b2e66744fab8923d6ee405a5e2). Dereplicator+ and network annotation propagation were used in conjunction for enhanced annotations. Although MolNetEnhancer is a workflow used for combining the results of CMN, Dereplicator+ and, NAP, this function did not work FBMN for annotating our results for the visualization of compound classes via Cytoscape at that time, due to server migration. Dereplicator+ also did not work for the FBMN annotation, revealing no hits. 

Workflows for Dereplicator+ and NAP can be found at (Task ID for NAP: 7a1f0ee11ab947408bcdf95f03a82e1b for the positive ionization mode and 12f645538264496c8a125a259f0ae810 for the negative ionization mode. For Dereplicator+, the task IDs were eef89c9cf95040628f6fb517ee64ccf7 for the positive ionization mode and eabb1d2b62124730ab10f3467310d975 for the negative ionization mode.

The task ID for the merged polarity network was 213e9cbd1ffb4735920662c63c96933.

The workflow for FBMN processing can be seen in the Supplementary Materials (Figure S1A). 

#### 2.7.4. Using SIRIUS as an In Silico Fragmentation Tool for Metabolite Annotation

mzXML files corresponding to the quality controls for each ecotype and both positive ionization modes were imported into SIRIUS 5.6.2. The peaks were classified and their MS^2^ fragmentation patterns tentatively annotated with the ZODIAC, CSI: FingerID, CANOPUS using ClassyFire and NPClassifier modules within SIRIUS 5.6.2 [68,70,71,72,73,74]. Default settings were used for the different modules, with Orbitrap being the instrument of choice, and MS^2^ queried within a 10 ppm error. For the positive ion mode, [M+H]^+^ and [M+H-H_2_O]^+^ adducts were used, with [M-H]^−^ being the adduct used in the negative ionization mode. All databases were used for tentative identifications. For the tentative identification, a combination of COSMIC and ZODIAC scores were both considered. A manual analysis of the matching substructures was conducted before a structural assignment was performed. If the fragmentation pattern did not match the structures proposed by CSI:FingerID, the matching of fragments from the proposed structures were considered when the class was assigned using CANOPUS. If the ZODIAC score was < 50%, no tentative identification was made. If the SIRIUS score was < 50% with no accompanying ZODIAC score, no identification was made. A combination of scores of >90% for SIRIUS and >70% for ZODIAC was used for screening [75,76,77]. 

#### 2.7.5. Cheminformatics Using ClassyFire for Compound Class Groupings 

For querying the data in cleaning up tentative metabolite names, and to identify compounds not named from both GNPS and SIRIUS, the Pubchem Identifier service (https://pubchem.ncbi.nlm.nih.gov/idexchange/idexchange.cgi accessed on 18 September 2023) was used to convert SMILES to InChiKeys for the batch compound classification tool in ClassyFire [73]. ClassyFire Batch Compound Classification (https://cfb.fiehnlab.ucdavis.edu/ accessed on 18 September 2023) was used to categorize InChiKeys into compound classes. SMILES without corresponding InChiKeys and InChiKeys not found in ClassyFire were not used for the metabolite annotations. Additionally, duplicate data were deleted after checking InChiKeys. InChiKeys are used for structural information instead of the SMILES format as they are standardized and have a fixed length, allowing for easy data processing [78].

## 3. Results

### 3.1. Untargeted Metabolomics: Analysis of D. dentatus Red and White Ecotypes 

Metabolomics analyses of the woody vine *D. dentatus* red and white ecotypes in natural communities has not been previously undertaken. Therefore, in this study, we aimed to explore and describe the metabolomic differences between these ecotypes on a global level, specifically focusing on the occurrence of secondary metabolites, such as polyphenols (i.e., flavonoids), terpenoids, alkaloids, and other compounds, in the *D. dentatus* red ecotype compared to the *D. dentatus* white ecotype to identify potential biofingerprints and/or differentially expressed compounds that may have been a potential therapy for erectile dysfunction in men. This study represents one of the initial efforts to investigate and present the descriptive metabolomic results for these two ecotypes. 

To investigate the dissimilarities in the metabolome between the *D. dentatus* red and white woody vine ecotypes, we conducted an untargeted metabolomics analysis using LC-MS/MS (liquid chromatography–tandem mass spectrometry). This allowed for the detection of 9562 tentative metabolite features that were further queried for exact and approximate matches in the databases of physiologically active substances in the literature and with bioinformatics servers used in untargeted metabolomics. 

Among these detected features, we observed 2664 tentative metabolite features with positive ionizations [M+H]^+^ and 2470 with negative ionizations [M-H]^−^ in the *D. dentatus* red ecotype. Similarly, in the *D. dentatus* white ecotype, we identified 2316 tentative metabolite features with positive ionizations [M+H]^+^. These findings indicate variations in the metabolite profiles between the red and white ecotypes of *D. dentatus*, as demonstrated by the differing numbers of detected tentative metabolite features in each ecotype.

We utilized the “Functional Analysis” module and the Gene Set Enrichment Assay (GSEA) tool within MetaboAnalyst 5.0 to better understand the metabolic differences between the two ecotypes. The purpose was to compare the tentative metabolite features (9562) obtained from our analysis. Using the GSEA tool, we searched for metabolite features in the annotated *A. thaliana* metabolite database that closely matched our mass charge ratio (*m*/*z*) features. This allowed us to find similarities with the Kyoto Encyclopedia of Genes and Genomes (KEGG) database and other bioinformatics servers used in untargeted metabolomics.

**Figure 3:**
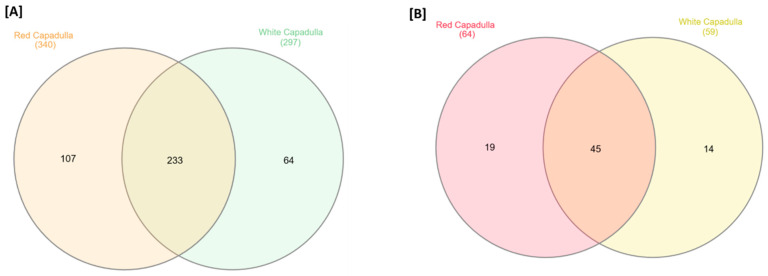
Venn diagrams illustrating the comparisons of tentative metabolite features and tentatively attributed pathways between the *D. dentatus* red and white ecotypes. Venn diagram (**A**) consists of the common and unique tentative features grouped in *D. dentatus* red- and white-ecotype woody vines. Venn diagram (**B**) consists of the tentatively attributed metabolite features in each woody vine *D. dentatus* ecotype using features from both ionization modes.

Upon conducting the global metabolomic analysis, we found a total of 637 putative metabolite features that matched with the reference database. Among these, 107 features (16.79%) were exclusive to the *D. dentatus* red ecotype, while 64 features belonged to the *D. dentatus* white ecotype (10.05%). Notably, approximately a portion % of these putative metabolite features, about (36.58%), were unique to the *D. dentatus* red or white ecotypes (Figure 3) [79]. 

Utilizing the abovementioned software tools and approaches, we successfully compared numerous metabolite features and identified the putative compounds that differed between the two ecotypes at levels 3 and 4 (Supplemental Tables S1–S7) [80]. This comprehensive analysis provided valuable insights into the metabolic variations within these ecotypes, further enhancing our understanding of their distinct characteristics. For further validation, MS^2^ data processing revealed 55 selected compounds composed of flavonoids, terpenoids, and some alkaloids at a level-2 annotation level (Table 1 and Table 2). 

**Figure 4:**
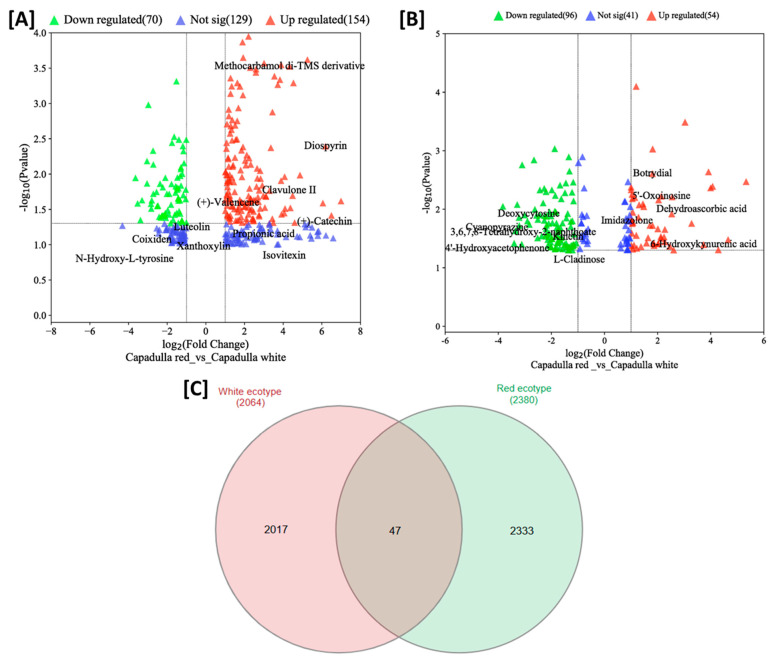
(**A**) Volcano plot from the mass spectrometry data demonstrates the magnitude and significance of *D. dentatus* red compared with the control (*D. dentatus* white) in (**A**) the positive ionization mode and (**B**) negative ionization mode. The horizontal dashed line shows where the *p*-value is 0.05 [−log_10_ (0.01) = 1.5], and the vertical lines show where the fold change is 2 [log_2_ (2) = 1] or 0.5 [log_2_ (0.5) = −1]. The two-fold change and *p*-value of 0.05 were used as the threshold cutoff. A total of 208 significantly upregulated, 166 downregulated, and 170 nonsignificant features were identified. (**C**) Venn diagram showing the repartition of the features obtained as statistical differences between *D. dentatus* red and white ecotypes combining both ionization modes. [79].

We utilized volcano plots to determine the tentative features in *D. dentatus* red that exhibited up- or downregulations compared to the *D. dentatus* white control in both positive and negative ionization modes. Several tentative *m*/*z* features deviated from the typical pattern within the volcano plot, indicating significant fold changes in regulating *D. dentatus* red and white ecotypes (Figure 4). To validate these tentative *m*/*z* features, we referred to the Omics Craft database module MetaboQuest (http://tools.omicscraft.com/MetaboQuest/, accessed on 10 June 2023) [79,80]. The analysis revealed compounds, such as Methocarbamol di-TMS derivative, Diosprin, Deoxycytosine, and others (Figure 4A). However, these putative compounds did not align with the biofingerprints of interest, particularly the polyphenolic compounds, such as catechin groups (Table 1 and Table 2). The presence of polyphenols is noteworthy as they possess antioxidant, anticancer, anti-inflammatory, cytotoxic, and antimicrobial properties, primarily due to their radical scavenging ability [81]. These findings suggest that a further exploration of polyphenolic compounds in *D. dentatus* red and white ecotypes may hold potential as a therapeutic response to erectile dysfunction. Their beneficial effects, attributed to their diverse bioactivities, make them a promising area of interest for potential therapeutic interventions.

### 3.2. Tentative Identification of the Significant Metabolites 

To compare the shared and unique tentative metabolite features among ecotypes, we utilized a Venn diagram [82]. For predicting the pathways to which the tentatively identified metabolite features belonged, we compared their accurate masses against the annotated metabolite database of *Arabidopsis thaliana* using the “Functional Analysis” module and the GSEA tool within MetaboAnalyst 5.0 (Figure 3). The GSEA tool searched for similar metabolite features in the annotated *A. thaliana* metabolite database and provided a ranked list of statistically significant metabolic pathways enriched in these metabolites. The list included corresponding *p*-values and adjusted *p*-values, indicating the significance level. GSEA employed a cutoff-free approach, searching for similarities between the metabolite features of the test species and *A. thaliana*. The resulting list ranked the metabolic pathways based on the similarity of metabolites and provided *p*-values derived from Kolmogorov–Smirnov tests [32,83]. 

The functional analysis tentatively attributed the characteristics of metabolites to 123 metabolic pathways within these two ecotypes. Out of these, 45 (95.94%) were present in both *D. dentatus* red and white ecotypes, whereas 19 (23.37%) were exclusive to the *D. dentatus* red ecotypes, and 14 (17.22%) were complete to the *D. dentatus* white ecotypes (Figure 3B).

### 3.3. Pathway Analysis of D. dentatus Ecotypes

A pathway analysis was performed to investigate the possible biomarkers within two separate ecotypes. This work aimed to elucidate the metabolic regulatory mechanisms that impacted the synthesis of diverse secondary metabolites and metabolic processes. A thorough analysis of the metabolic pathways was performed to identify the difference between the red and white ecotypes of *D. dentatus.* Databases, such as KEGG and HMDB, were used to analyze at the obtained biomarkers.

The research utilized a range of techniques for visualizing and analyzing the data. The data were shown using a scatter plot, and an enrichment analysis was performed using Fisher’s exact test. Furthermore, a topological study was conducted to examine the out-degree centrality. These methods were selected based on their statistical significance and capacity to evaluate the influence of routes.

**Figure 5:**
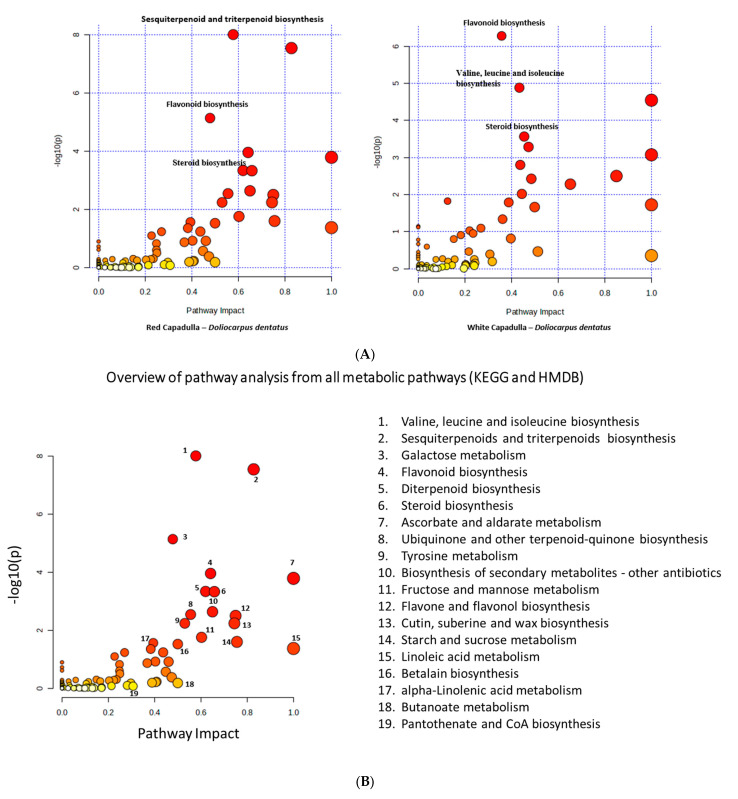
(**A**) Overview of pathway analysis from all metabolic pathways (KEGG and HMDB) for *D. dentatus* red and white ecotypes. All the matched pathways were classified by *p*-values (y-axis) from the pathway enrichment analysis and pathway impact values (x-axis) from the pathway topology analysis. The node size exhibits the effect of impact values. The node colors exhibit different *p*-values. (**B**) Integrated pathway activity profile of significant (*p* ≤ 0.05) metabolite features using both *D. dentatus* ecotypes linked to biosynthetic pathways as analyzed by MetaboAnalyst 5.0 (GSEA algorithm) using default parameters and the *Arabidopsis thaliana* pathway as the library. The color gradient from yellow to red and size of dot indicate statistical significance and impact of pathways.

The red ecotype of *D. dentatus* showed a statistical significance (*p* ≤ 0.05) in the pathways related to the formation of sesquiterpenoids and triterpenoids, galactose metabolism, flavonoids, steroids, flavones, and flavonols. Additional data further substantiated these findings, demonstrating statistical significance values ranging from *p* = (0.000 to 0.01586) for the pathways mentioned above. The red ecotype of *D. dentatus* was statistically involved in the biosynthesis of sesquiterpenoid and triterpenoid, the biosynthesis of flavonoid, and galactose metabolism. The significance levels for these pathways ranged from *p* = (0.03 to 0.58). In contrast, the influence of limonene and pinene degradation, as well as the production of anthocyanin, an isoquinoline alkaloid, tropane, piperidine, and pyridine alkaloid, was shown to be relatively insignificant, as illustrated in Figure 5A and Supplemental Table S9.

Regarding the *D. dentatus* white ecotype, the metabolic pathways that exhibited the most importance were flavonoid biosynthesis, valine, leucine, and isoleucine biosynthesis. The statistical significance of these routes ranged from *p* = (0.0000 to 0.00046416). Additionally, the impact of these pathways varied, with significance levels ranging from *p* = 0.08 to 0.36. In contrast, the degradation of valine, leucine, and isoleucine; inositol phosphate metabolism; and tryptophan metabolism had reduced impacts, as depicted in Figure 5B and Supplementary Table S8. Given the previous literature on the *D. dentatus* species, it is plausible that these metabolic pathways exert substantial effects and can influence the taxonomic classification at distinct hierarchical levels [84]. 

In the context of biochemistry and nutrition, the interactions between compounds often yield various health benefits. These interactions are multifaceted and can result in enhanced outcomes. For example, antioxidants, such as epicatechin methyl gallate, catechin gallate, and proanthocyanidin A2, can collaborate to neutralize harmful free radicals more effectively, extending protection against oxidative damage [85]. Additionally, some compounds can improve the absorption and utilization of others, increasing the availability of essential nutrients, such as vitamins and minerals. Metabolic interactions can also occur, enhancing the effectiveness or duration of specific compounds [86]. Furthermore, compounds interacting with the same cellular pathways can regulate cellular processes cooperatively, leading to improved cell function and the modulation of inflammation. Nutrient synergy is another aspect where certain compounds are more effective when consumed together, while balancing effects can mitigate adverse outcomes of specific compounds when consumed alone [87].

These interactions are intricate and context dependent, influenced by factors, such as the types of compounds involved, their concentrations, individual genetics, and overall dietary and lifestyle choices. Researchers continually explore these complex dynamics to better comprehend how compounds collectively impact health and well-being, especially in the case of ED.

### 3.4. Principal Component Analyses of D. dentatus Red and White Ecotypes

**Figure 6:**
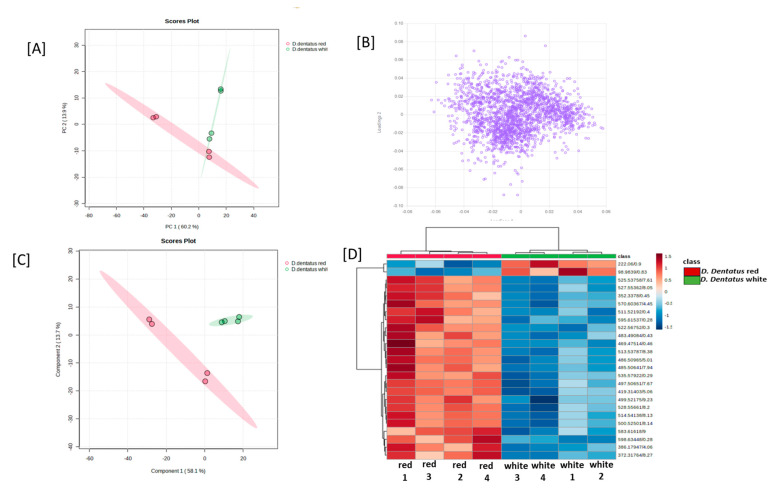
(**A**) Principal component analysis score plot reflecting the visualization of the relationship among the samples in terms of groupings, trends, or outliers, and showing differences between *D. dentatus* red and white ecotypes along the x−axis (PC1) and y−axis (PC2) from positive ionization data. Principal components 1 and 2 explain 60.2% and 13.9% of the variance, respectively. (**B**) Loading plot describing the influence of variables on sample segregation. (**C**) Partial least-squares discriminant analysis (PLS-DA) was applied to differentiate between *D. dentatus* red and white ecotypes. (**D**) Hierarchical cluster analysis (HCA), computed based on the top 25 statistically significantly different metabolite features, grouped the samples into two groups and by intensities. The color scale indicates the relative intensity of each metabolite, and the color-coded dendrogram on the top of the HCA plot indicates the relationship among the samples.

**Figure 7:**
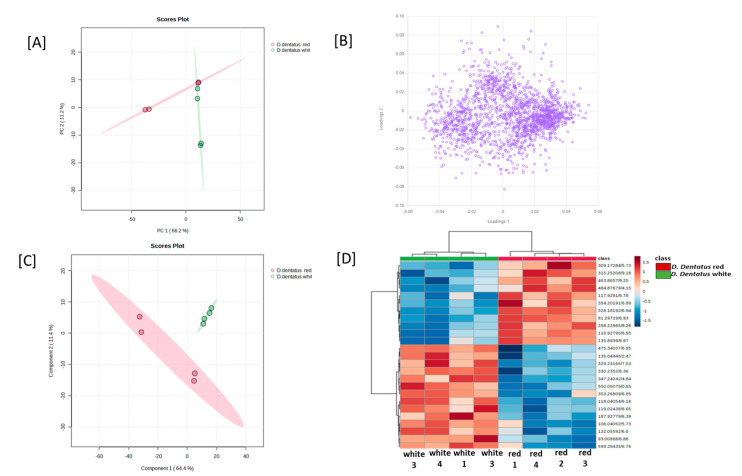
(**A**) Principal component analysis score plot reflecting visualization of the relationship among the samples in terms of groupings, trends, or outliers, and showing differences between *D. dentatus* red and white ecotypes along the x−axis (PC1) and y−axis (PC2) from negative ionization data. Principal components 1 and 2 explain 66.2% and 11.2% of the variance, respectively. (**B**) Loading plot describing the influence of variables on sample segregation. (**C**) Partial least-squares discriminant analysis (PLS-DA) was applied to differentiate between *D. dentatus* red and white ecotypes. (**D**) Hierarchical cluster analysis (HCA), computed based on the top 25 statistically significantly different metabolite features, grouped the samples into two groups and by intensities. The color scale indicates the relative intensity of each metabolite, and the color-coded dendrogram on the top of the HCA plot indicates the relationship among the samples.

Principal component analysis was conducted on all the samples based on the LC-MS data. The overall relationship among the samples was assessed by PCA using all the tentative metabolite features identified for all the samples; on a PCA plot, samples that were spatially close to each other had more similar metabolite profiles. The PCA generated two distinct clusters, each cluster corresponding to an ecotype of *D. dentatus*. The first two principal components, PC1 and PC2, explained 60.3% and 13.9%, respectively, of the variability in the positive ionization mode. In contrast, 67% and 11% in the negative ionization mode of the total variability among the samples and the clusters that corresponded to the *D.dentatus* white ecotype and *D. dentatus* red ecotype, respectively, were closer to each other, indicating overall similarities in their metabolite profiles (Figure 6 and Figure 7). The same pattern of relatedness was also observed in a dendrogram based on the Euclidean distance, on which the *D. dentatus* red and *D. dentatus* white ecotypes were grouped in the same clade (Figure 6 and Figure 7).

The metabolic profiles of *D. dentatus* red and white ecotypes were examined with OPLS-DA to probe for metabolite features that separated the samples into distinct clusters corresponding to the ecotypes (Figure 6 and Figure 7; Supplemental Figure S1B). The accumulation of the top 15 statistically significant metabolite features that separated the samples into the two main PLS-DA clusters was mainly influenced by the relative intensity of the sample and the ecotypes (Figure 6 and Figure 7; Supplemental Figure S1B). However, a close inspection of the relative abundance of some of the top statistically significant metabolite features indicated that species and treatment influenced their abundance (Figure 6 and Figure 7). For instance, the relative abundance of the flavonoids was significantly higher in the *D.dentatus* red ecotype than in the *D. dentatus* white ecotype (Figure 7; Supplemental Figures S2 and S5). The secondary metabolites putatively identified, such as alkaloids and terpenoids, were comparable between the ecotypes (Figure 5; Supplemental Figures S3 and S4).

### 3.5. Metabolite Annotation and Compound Class Contribution Using Tandem Mass Spectrometry Data

**Figure 8:**
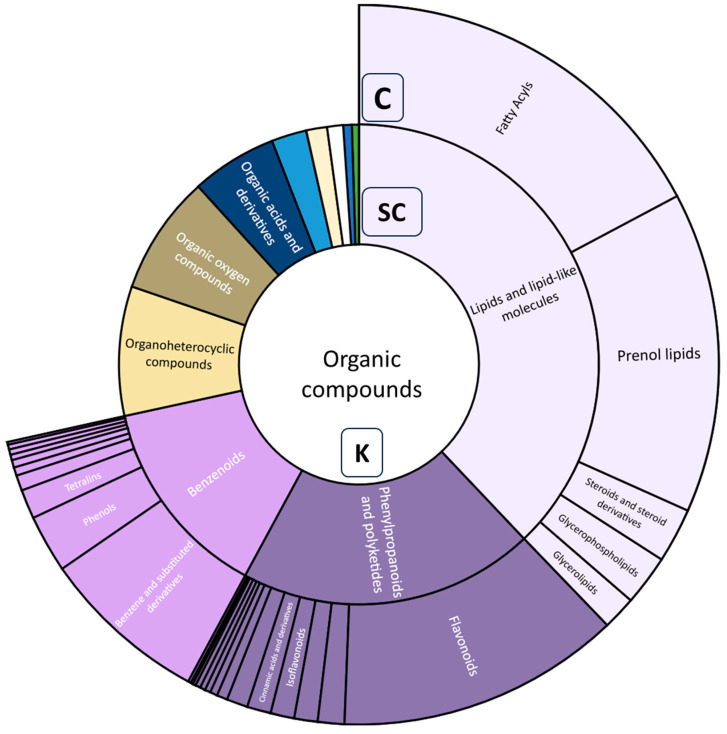
Sunburst plot of the different class levels of the nodes annotated by ClassyFire by compounds from both positive and negative ionization modes from both red and white *D. dentatus* ecotypes. ClassyFire summarizes the compounds according to the kingdom level (K), superclass level (SC), and class level (C). Benzenoid, phenylpropanoid, and polyketide, and lipids and lipid-like molecule superclasses dominate in contributions of annotated compounds.

We annotated 847 compounds ranging from levels 2 to 4 by combining the usage of GNPS, MS DIAL, and SIRIUS, and compounds without fragmentation data (MS^1^). Approximately 12% of compounds annotated did not have fragmentation data and were placed at annotation level 4. In pooling contributions of red and white ecotypes, with ClassyFire used to categorize the compounds, most of the superclass of compounds fell into benzenoids, lipids, and lipid-like molecules, and phenylpropanoids and polyketides at 13.7, 37.9, and 20%, respectively (Figure 8). The phenolic compounds screened for therapeutic potential were majorly distributed in these categories. Although alkaloids were a superclass of interest, they were the second lowest contributor to the annotated compound pool at 0.6%.

**Figure 9:**
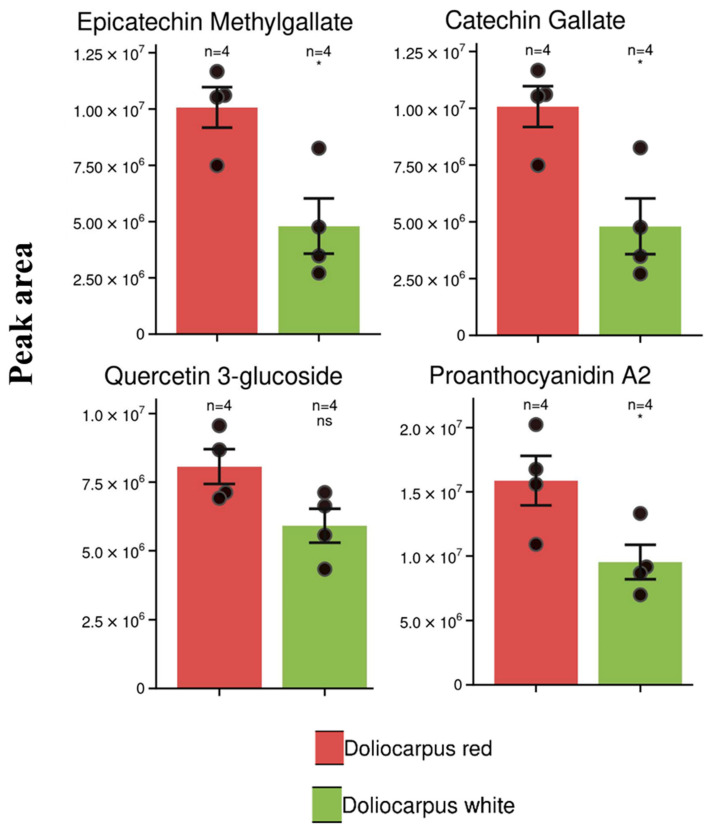
Flavonoid biomarkers of *D. dentatus* red and white ecotypes’ normalized peak areas (mean ± SE) of the top metabolite features identified by PLS-DA. Within each metabolite, the intensity is dependent on the combination of ecotypes (ANOVA red ecotype × white ecotype (control interaction)): proanthocyanidin A2 *p* < 0.04, epicatechin methyl gallate *p* < 0.015, and catechin gallate *p* < 0.015. “*” and “ns” represent statistical significance and not significant, respectively.

In investigating phenylpropanoids and polyketides, the flavonoid class dominated the pool at 63.9%, followed by macrolides and monologues at 5.9%, with equal contributions of cinnamic acids and derivatives, isoflavonoids, and linear 1,3-diarylpropanoids at 5.3% each (Figure 9). Under lipids and lipid-like molecules, the fatty acyl class contributed most to the pool at 45.5%, followed by prenol lipids at 38%, and steroids and steroid derivatives at 6.54%. Terpenoids, another category of interest for therapeutic potential, fell into prenol lipids.

With respect to the biosynthetic pathways, using the mummichog algorithm under MetaboAnalyst 5.0 for the initial screening of MS^1^ data, the pathway analysis results aligned with the annotated compounds, especially with high contributions of compounds belonging to both flavonoid and terpenoid biosynthesis pathways (Figure 5A,B).

## 4. Discussion

The Orbitrap used in the present work had a high resolving power and excellent mass accuracy, precise mass measurements, and broad analyte coverage, providing greater confidence in identification, especially for complex matrices [88,89,90,91,92]. Moreover, the full-scan mode allowed the recording of unlimited compounds, which made it feasible for the retrospective analysis of any potential compounds of interest. The Orbitrap HRMS can also reduce the method cost and development time by not having to purchase multiple standards or in performing manual method set-ups. To date, Orbitrap HRMS has been extensively used in mass spectrometry metabolomics base research for targeted and non-targeted analyses. However, LC-MS has some drawbacks, including high instrumentation costs, complex data analysis, time-consuming data processing, and challenges related to sample preparation and matrix effects. Therefore, the metabolite annotation was performed by spectral library matching as a first step. Several candidates of *D. dentatus* red and white putative metabolites were then shortlisted. Those that showed identical names from KnapSack [33] (Shinbo, Nakamura et al. 2006), KEGG [34] (Kanehisa, Sato et al. 2019), the Plant Metabolic Network using the PlantCyc database (https://plantcyc.org, accessed on 10 June 2023) [35], MetaboQuest [36], SciFinder [37] and PubChem [38], Global Nature Production Social Molecular Networking (GNPS), SIRIUS, and MS Dial were used to query fragments for putative identifications [3,15]. Then, peak confirmation was performed with Xcalibur 4.1 having a mass error of ±10 ppm to determine whether these metabolites were present in the methanol extracts of *D. dentatus* red and white ecotypes.

### 4.1. Untargeted Metabolomics Analysis Revealed Metabolites Present in Different Ecotypes of Doliocarpus Dentatus

Secondary metabolites comprise organic chemicals that plants, fungi, and microbes synthesize. These compounds play diverse roles in ecological and adaptive processes. Plants provide several ecological functions, including herbivore defense, pollinator attraction, and the facilitation of environmental adaptations [93,94]. The secondary metabolites, including flavonoids, terpenoids, and alkaloids, possess significant value in traditional medicine, pharmaceuticals, perfumery, and flavoring.

The metabolites identified in the different *D. dentatus* ecotypes were diverse, with some reported to have therapeutic benefits due to their varied bioactivities. Using the LC-MS/MS analysis, we found polyphenolic, terpenoid, and alkaloid metabolite classes, with polyphenols (flavonoids) being the most statistically significant and relatively abundant between the red and white ecotypes.

There was a statistically significant difference between the polyphenols in the *D. dentatus* red ecotype and the *D. dentatus* white ecotype (Figure 9). Flavonoids, such as epicatechin methyl gallate, catechin gallate, and proanthocyanidin A2, significantly differed in the *D. dentatus* red ecotype compared to the *D. dentatus* white ecotype based on the integrated peak areas. Epicatechin methyl gallate, catechin gallate, proanthocyanidin A2 and proanthocyanidin B2 isomers, quercetin 3 glycoside, apigenin, (+)-Catechin, leucocyanidin, anthocyanidin 3-O-beta-D-sambubioside, and resveratrol are different types of bioactive compounds commonly found in *D. dentatus* (see Table 1 and Table 2, Supplemental Tables S1–S7). These compounds belong to other classes of natural products and possess diverse biological activities. Epicatechin methyl gallate, a bioactive compound found in various plant sources, exhibits antioxidant and anti-inflammatory properties that have attracted significant scientific interest due to its potential health-promoting properties [95]. One of its notable benefits is its ability to reduce oxidative stress and inflammation in cells.

#### 4.1.1. Flavonoids

Flavonoids are a class of secondary metabolites mainly composed of a benzopyrone ring that contains phenolic or polyphenolic groups at various locations [96] The biosynthesis of phenols relies on two pathways: the shikimic acid and malonic acid pathways [97,98]. These compounds are predominantly present in various botanical sources, such as fruits, herbs, stems, grains, nuts, vegetables, flowers, and seeds [99,100]. The therapeutic efficacy and biological activity of these various plant components are attributed to the presence of bioactive phytochemical substances [101]. Flavonoids may offer antioxidant, anti-inflammatory, anticancer, cardiovascular, and neuroprotective benefits [102], which may contribute to reducing several sexual dysfunctions, such as low sexual desire, erectile dysfunction, an inability to achieve orgasm, and premature ejaculation, which are relatively common in men, even at a young age [103,104,105,106,107,108].

Moreover, the research indicates that incorporating better lifestyle behaviors might enhance erectile function. Specifically, implementing dietary modifications and the augmentation of nutritional antioxidant consumption appear to be the most encouraging and economically viable strategies for addressing erectile dysfunction [109,110]. The consumption of several antioxidant-rich foods, such as pomegranate juice, coffee, wine, nuts, and ginseng, has been associated with lower ED prevalence [111,112,113,114,115,116].

In general, it has been proposed that incorporating a healthy diet, which emphasizes consuming fruits and vegetables while limiting animal protein intake, can potentially improve symptoms of ED. This dietary approach reduces glucose and lipid metabolism, enhances antioxidant defenses, and elevates arginine levels. Consequently, these effects contribute to increased nitric oxide (NO) activity, ultimately improving erectile function [116,117,118,119].

The justification for employing antioxidants to mitigate the symptoms of erectile dysfunction is derived from the discovery that ED frequently occurs before the manifestation of cardiovascular (CV) conditions. Antioxidant chemicals have gained recognition for their advantageous impact on mitigating cardiovascular risk, implying their potential to resolve symptoms of erectile dysfunction [120].

Catechin gallate, found in *D.dentatus*, possesses intriguing properties that have attracted scientific attention (see Table 1 and Table 2). Similar to other catechins, it exhibits antioxidant activity and potential health benefits. Its antioxidant properties help reduce oxidative stress and prevent chronic diseases associated with free radicals, such as cardiovascular disease [121,122]. Catechin gallate also exhibits anti-inflammatory effects, mitigating inflammation and potentially reducing the risk of chronic inflammatory diseases. Additionally, it may positively impact cardiovascular health by improving lipid profiles, lowering blood pressure, and enhancing vascular function [123,124,125]. It has antioxidant properties, which help protect cells from damage caused by harmful bodily molecules. (+)-Catechin may have several health benefits, including promoting cardiovascular health, reducing inflammation, and potentially aiding in weight management [126,127,128].

Proanthocyanidin B2 is a specific type of proanthocyanidin, which is a type of flavonoid found in various plants. Proanthocyanidins are known for their antioxidant properties and are commonly found in foods, such as fruits, vegetables, nuts, and seeds, and certain beverages, such as red wine and tea [129]. Proanthocyanidin B2 has been studied for its potential health benefits. It exhibits antioxidant activity, helping to neutralize harmful free radicals in the body [130]. It also has anti-inflammatory properties, which may be beneficial in reducing inflammation-related conditions. Proanthocyanidin B2 may contribute to cardiovascular health by promoting healthy blood vessel function and reducing the risk of blood clot formation.

Additionally, it has been investigated for its potential anticancer properties, as it may help inhibit the growth of cancer cells [131]. Proanthocyanidin A2 is a flavonoid subclass found in plants recognized for their antioxidant capabilities. It is generated by polymerizing flavan-3-ol monomers and is distinguished by its condensed tannin structure [129]. Proanthocyanidins, especially Proanthocyanidin A2, have been linked to various health benefits, including antioxidant activity, cardiovascular health, anti-inflammatory properties, skin health, and dental health [132].

Proanthocyanidins can neutralize dangerous free radicals, promote healthy blood flow, lower blood pressure, and enhance lipid profiles [133]. Quercetin 3-glucoside, also known as isoquercitrin, is a natural plant compound found in fruits, vegetables, and herbs (see Table 1 and Table 2). It belongs to the glycoside class and has antioxidant properties that protect cells from free radical damage. Quercetin 3-glucoside also has anti-inflammatory effects, which may help reduce inflammation and manage inflammation-related conditions [134,135]. It has been studied for its potential cardiovascular benefits, such as improving blood vessel function, lowering blood pressure, and inhibiting blood clot formation. Quercetin 3-glucoside also modulates the immune system, potentially supporting immune function and defense against infections [136]. Its antioxidant and anti-inflammatory properties may promote healthy skin by protecting against oxidative stress and inflammation [137,138].

Apigenin is a natural flavonoid molecule in many plants with antioxidant, anti-inflammatory, and anticancer activities [139]. Its prevalence in plant-based meals, such as parsley, celery, and chamomile tea, contributes to its potential health advantages [140]. Apigenin is an antioxidant that neutralizes damaging free radicals and lowers oxidative stress associated with chronic illnesses, such as cancer, cardiovascular disease, and neurological disorders [139,141]. It also possesses anti-inflammatory qualities, which aid in reducing inflammation and discomfort. Apigenin has been found in studies to cause cell cycle arrest and death in cancer cells, potentially reducing their growth and spread [141,142]. Apigenin has been studied for its potential for cancer prevention and therapy, including applications in breast, colon, prostate, and lung malignancies. It has also been examined for its potential to improve cardiovascular health, cognitive function, and immune system performance [143,144]. Leucocyanidin is a bioactive compound in plants belonging to the flavan-3-ol flavonoid family. It has potent antioxidant capabilities that protect cells from oxidative damage [145]. Moreover, leucocyanidin has anti-inflammatory properties that may help minimize the risk of chronic inflammatory illnesses. It also benefits cardiovascular health by promoting healthy blood vessels and heart function [146]. While further study is needed, leucocyanidin’s antioxidant, anti-inflammatory, and cardiovascular effects make it a promising molecule for overall well-being and disease prevention.

Anthocyanidin 3-O-beta-D-sambubioside is a unique anthocyanin pigment that contributes to the vibrant colors of fruits, vegetables, and flowers. Its attachment to beta-D-sambubioside makes it unique, contributing to the vibrant colors in plant-based foods and attracting pollinators for plant reproduction. Anthocyanidin 3-O-beta-D-sambubioside offers potential health benefits due to its antioxidant properties, which neutralize harmful free radicals in the body [147,148,149,150]. These free radicals can cause cellular damage and contribute to chronic diseases, such as cardiovascular disease and cancer [148,151]. Additionally, the research suggests that anthocyanidin 3-O-beta-D-sambubioside may have anti-inflammatory effects, modulating inflammatory responses and reducing the risk of chronic diseases [147,152]. The presence of anthocyanidin 3-O-beta-D-sambubioside in fruits and vegetables makes them visually appealing and potentially beneficial for health.

The bioactive compounds discussed previously, such as epicatechin methyl gallate, catechin gallate, (+)-Catechin, proanthocyanidins, quercetin 3-glucoside, and apigenin, offer various health benefits, especially for ED due to their antioxidant and anti-inflammatory properties [153]. These compounds act as antioxidants, neutralizing harmful free radicals and reducing oxidative stress in cells, which helps protect against cellular damage and supports overall well-being. Additionally, they show potential in promoting cardiovascular health by improving lipid profiles, lowering blood pressure, and enhancing blood vessel function, which may reduce the risk of ED.

#### 4.1.2. Alkaloids

Alkaloids are a class of organic molecules characterized by nitrogen atoms with elemental properties, often found in many organisms, including plants, fungi, and mammals [154,155]. The biosynthesis of alkaloids relies on the Shikimic acid and Malonic acid pathway [156,157]. These important secondary metabolites have been recognized for their medicinal potential [158]. These compounds are categorized into several groups based on their biosynthetic precursor and heterocyclic ring system. These groups include indole, piperidine, tropane, purine, pyrrolizidine, imidazole, quinolozidine, isoquinoline, and alkaloids [159]. Alkaloids may hinder the initiation of diverse degenerative ailments through their ability to scavenge free radicals or bind with catalysts involved in oxidative reactions [160].

The secondary metabolites, such as trigonelline, xanthurenic acid, thiazole, and raphanin, are all present in *D. dentatus,* of which their relative abundance is higher in the *D. dentatus* white ecotype. At the same time, 4-Methylelletierine, N–Methylelletierine, Nicotinamide, Stachydrine, and Acetylbrowniine were higher in the *D. dentatus* red ecotype (see Supplemental Tables S1–S7).

Trigonelline exhibits antioxidant and anti-inflammatory properties, and may aid blood sugar regulation and metabolic health [161]. Xanthurenic acid is involved in amino acid metabolism and is linked to regulating insulin levels and glucose metabolism [162]. Thiazole shows diverse biological activities and has garnered interest in medicinal chemistry [163]. Methylelletierine and N-Methylelletierine possess anthelmintic properties, which are beneficial for combating parasitic infections. Nicotinamide and stachydrine are associated with anti-hypertensive effects and affect blood pressure regulation [164]. Acetylbrowniine offers anti-inflammatory properties [165], while rapanin, derived from radishes, has potential antioxidant and anticancer effects [166]. These compounds interact with biological systems in distinct ways, offering a range of potential health benefits; however, further research is needed for a comprehensive understanding of their effects.

Secondary metabolites 4-Methylelletrine, N-Methylelletrine, Nicotinamide, Stachydrine, Trigonelline, and Acetylbrowniine have not been subjected to comprehensive investigations as potential therapeutic interventions for erectile dysfunction. Although several compounds may possess features that might potentially impact the aspects associated with ED, such as regulating blood pressure, cardiovascular well-being, and blood sugar control, there is currently insufficient data to substantiate their efficacy as primary therapies for ED.

#### 4.1.3. Terpenoids

Terpenoids are a large and diverse class of structurally secondary metabolites derived from five-carbon isoprene units naturally occurring in many plants [167]. The biosynthesis of the mevalonic acid pathway relies on the shikimic acid and malonic acid pathway [156,157,168]. Terpenoids differ due to their functional groups and C10-unit hydrocarbon backbone. These are further categorized based on the number of carbocyclic rings, such as monoterpenes (limonene, carvone, (1R)-(-)-Nopol and carveol), diterpenes (the retinoids), and tetraterpenes (α- and β-carotene, lutein, lycopene, zeaxanthine, betulinic acid, betulin, and cryptoxanthine) [169,170]. These subclasses may have anti-inflammatory, anti-cancer, antioxidant, anti-inflammatory, and antimicrobial properties [171]. Terpenoids are also essential for plant growth and development, impart flavor, aroma, and color to plants’ foliage, flowers, and fruits [172,173], and defend them against insects, herbivores, and fungal diseases [174].

Secondary metabolites, such as alpha-pinene, ascaridole, (1R)-(-)-Nopol, 3-(beta-D-glucopyranosyloxy)-2-methyl-4H-Pyran-4-one, 4-(2,6,6-trimethyl-2-cyclohexen-1-yl)- 2-Butanone, farnesol, betulinic acid, betulonic acid, sumaresinolic acid, betulin, and saikogenin D, are all present in *D. dentatus,* of which their relative abundance is comparable between *D. dentatus* red and white ecotypes (see Table 1 and Table 2 and Supplemental Tables S1–S7).

These compounds have diverse bioactive characteristics, from their potential use in medicine to their roles as natural antibacterial agents, antioxidants, and enhancers of olfactory and gustatory sensations. Nevertheless, it is imperative to highlight that the precise bioactivities and possible health advantages of these compounds are currently being investigated, and their efficacy may differ based on the particular circumstances in which they are utilized, thus requiring additional investigations.

The current body of research on the influence of terpenoids on ED is still constrained. However, it is important to highlight that terpenoids, naturally present in various plants, can indirectly impact aspects related to ED. Terpenoids with anti-inflammatory and antioxidant characteristics have promising potential in enhancing vascular health and mitigating oxidative stress, pertinent factors in ED. Specific terpenoids have the potential to facilitate vasodilation, which can enhance blood circulation and thus have an indirect impact on improving erectile function [175]. Moreover, several terpenoids, recognized for their anxiolytic and stress-alleviating properties, can alleviate psychological elements contributing to ED. Additional investigations are necessary to clarify the exact processes and effectiveness of terpenoids in managing ED, considering their significant potential in treating this complex ailment.

#### 4.1.4. Therapeutic Compounds and Possible Synergistic Effects

The compounds discussed in this research demonstrated many synergistic interactions, particularly in the domains of fragrance, possible therapeutic effects, and anti-cancer capabilities [176]. The combination of alpha-pinene and (1R)-(-)-Nopol in essential oils creates pleasant scents that contribute to the overall olfactory attractiveness of these oils. The potential exists for a synergistic interaction between farnesol and saikikogenin D, resulting in enhanced anti-inflammatory and antibacterial activities, hence increasing their therapeutic efficacy [177]. In the context of anticancer activities, it has been shown that compounds, such as betulin, betulonic acid, betulinic acid, and sumaresinolic acid, may potentially collaborate to produce favorable outcomes. The degree of synergy between 3-(beta-D-glucopyranosyloxy)-2-methyl-4H-Pyran-4-one and 4-(2,6,6-trimethyl-2-cyclohexen-1-yl)-2-Butanone is contingent upon the specific applications in which they are utilized [178].

In the domain of ED, secondary metabolites, such as flavonoids, alkaloids, terpenoids, and other phytochemicals in whole-plant extracts have been observed to facilitate favorable interactions, presenting potential advantages [179]. These interactions may augment blood circulation, mitigate inflammation, and increase vascular and sexual well-being. The research shows that green tea polyphenols, particularly epigallocatechin gallate (EGCG), have antioxidant properties that may reduce oxidative stress in the body, which is known to contribute to blood vessel damage and impaired blood flow, factors often linked to ED [95]. Yohimbine is an alkaloid found in yohimbe bark, and it is known to have vasodilatory effects, which means it can relax and widen blood vessels, improving blood circulation. In the context of ED, an increased blood flow to the penis is essential for achieving and maintaining an erection [180]. Ginseng contains various terpenoids that may enhance nitric oxide production. Nitric oxide is a signaling molecule that plays a crucial role in dilating blood vessels and promoting blood flow to the penis, which may improve erectile function [116,117,118,119].

The synergistic effects of polyphenol-rich extracts derived from sources, such as green tea, dark chocolate, and berries; alkaloids present in yohimbine and ginseng; and terpenoids sourced from herbs, such as ginkgo biloba and ginseng, may potentially collaborate to enhance blood circulation, induce vasodilation, and augment nitric oxide synthesis, thereby potentially contributing to the amelioration of erectile dysfunction [181,182,183].

Moreover, the aforementioned phytochemical interactions have the potential to offer antioxidant assistance, hence mitigating the detrimental effects of oxidative stress on blood vessels and the consequential impairment of erectile function. The potential enhancement of sexual health and the alleviation of symptoms related to ED can be achieved through the synergistic actions of polyphenols, alkaloids, and terpenoids derived from various sources.

Reactive Oxygen Species (ROS) play important roles in various diseases, including cancer, obesity, and ED [184,185,186,187]. ROS can negatively impact the function of blood vessels in the penis, leading to vascular dysfunction. Excessive ROS levels can cause oxidative damage to the endothelial cells that line the blood vessels, reducing their ability to produce and release nitric oxide (NO). NO plays a crucial role in vasodilation, essential for achieving and maintaining an erection [187]. ROS-induced endothelial dysfunction reduces NO bioavailability, resulting in inadequate blood flow to the penile tissues and erectile dysfunction [188,189].

Moreover, ROS can directly interact with and deactivate NO, further exacerbating the impairment of NO signaling in erectile function [190]. NO is a vital signaling molecule that induces smooth muscle relaxation in the penile tissue, facilitating the dilation of blood vessels and the influx of blood necessary for an erection [188]. Increased ROS production can lead to the formation of reactive nitrogen species (RNS), which react with NO to create peroxynitrite, a highly reactive compound that impairs NO-mediated vasodilation [191].

The penile tissue is susceptible to oxidative damage caused by ROS. Excessive ROS levels induce oxidative stress, leading to lipid peroxidation, protein oxidation, and DNA damage within penile cells. These harmful effects disrupt the normal cellular function and integrity of the penile tissue, impairing its ability to achieve and sustain an erection [192]. Furthermore, ROS-induced oxidative stress can trigger inflammatory responses in the penile tissue. Chronic inflammation can activate fibroblasts and promote the deposition of extracellular matrix proteins, leading to fibrosis [193]. Penile fibrosis involves an excessive accumulation of collagen and other fibrous components, resulting in structural changes and the stiffness of the erectile tissue, further contributing to erectile dysfunction [194].

The detrimental effects of ROS-induced damage on erectile function are multifaceted. Excessive ROS production can impair vascular function, disrupt NO signaling, cause tissue oxidative damage, promote inflammation and fibrosis, and contribute to age-related erectile dysfunction. Understanding the mechanisms through which ROS influences erectile function can guide the development of therapeutic strategies to mitigate oxidative stress and preserve erectile health. However, further research is needed to understand their effects and specific applications fully.

Plants exhibit an impressive capacity for synthesizing a wide array of organic compounds. Upon conducting an LC-MS/MS analysis on Guyana Capadulla ecotypes, we identified a diverse range of secondary metabolites, such as alkaloids, terpenoids, fatty acids, phenylpropanoids, organic acid, polyketides and gallotannins, lignans, and flavonoids. These organic compounds are believed to have evolved as a part of plant defense mechanisms against various environmental challenges, including pests, diseases, and droughts [195]. Notably, among the identified metabolites that were found in both ecotypes in more than one sample were, salsolinol (alkaloid), lutein (carotenoids), and lyoniside (lignans: iridoid glycosides) (Supplemental Tables S4–S6). Salsolinol, found in Guyanese Capadulla, is a natural compound in the isoquinoline alkaloid class that is a combination of dopamine and acetaldehyde [196]. The research focuses on its effects on addiction and reward pathways in the brain, particularly its role in reinforcing drug-seeking behavior and addiction to substances, such as alcohol, nicotine, and opioids [197]. Salsolinol’s interactions with neurotransmitter systems in the brain may contribute to the pleasurable effects of drugs and the development of addictive behaviors. Additionally, it has been studied for its potential neurotoxic effects, leading to oxidative stress and damage to neurons, which can be relevant to neurodegenerative diseases [196]. However, further research is needed to fully understand its mechanisms and potential therapeutic applications.

In particular, green leafy vegetables, such as spinach, kale, and broccoli, contain lutein, a naturally occurring carotenoid pigment [198]. It is an effective antioxidant that protects cells from the damage caused by risky free radicals, which can result in chronic diseases and aging [199]. Because it is a vital component of the macular pigment, which serves as the macula’s protective layer, lutein is also crucial for maintaining eye health. This pigment protects retinal tissues from ultraviolet- and high-energy blue-light damage, reducing the risk of age-related macular degeneration (AMD) and cataracts. A diet high in lutein may be linked to a better cognitive performance and a lower risk of cognitive decline in older people, according to the research on the possible effects of lutein on cognitive function [200]. To conclusively link lutein intake and cognitive health, additional research is necessary. A diet high in fruits and vegetables, primarily one high in lutein, can support general health and the maintenance of healthy eyes.

Specific plant sources contain a naturally occurring flavonoid glycoside called lyoniside, which has potent anti-inflammatory and antioxidant properties [201], and is putatively identified in Guyanese Capadulla. It functions as an antioxidant to protect cells from the damage of harmful free radicals, which can accelerate aging and lead to chronic diseases. Furthermore, lyoniside has demonstrated its potential as a neuroprotective compound, protecting neurons and promoting brain health [202]. Although lyoniside research is still in its early stages, the novel findings indicate that it has a high potential for therapeutic use, particularly in treating inflammatory and oxidative stress-related diseases.

### 4.2. Several Metabolites Are Ecotype Specific

We described ecotype-specific metabolites as those present in only one ecotype (Supplemental Tables S2 and S3).

For instance, (-)-Epigallocatechin, anthocyanidin 3-O-beta-D-sambubioside, and naringenin (Supplemental Tables S2), which were present only in the *D. dentatus* red ecotype, are three bioactive compounds with significant potential in various fields. Epigallocatechin, found in green tea, exhibits antioxidant and anti-inflammatory properties, contributing to its health benefits [203]. Anthocyanidin 3-O-beta-D-sambubioside, a plant flavonoid, presents antioxidant and anticancer properties, potentially aiding disease prevention [204]. Naringenin, commonly found in citrus fruits, has anti-inflammatory and cardiovascular health-promoting effects, making it valuable for overall well-being [205,206]. These compounds hold promise for further research and applications in medicine, nutrition, and functional foods.

Quercetin and myricetin, present only in the *D. dentatus* white ecotype, are two flavonoids renowned for their high bioactive characteristics and multiple health benefits [207]. Quercetin is present in various fruits and vegetables, including apples, onions, and berries, and is known for its antioxidant, anti-inflammatory, and immune-modulating properties. It has been examined for its ability to reduce the risk of chronic illnesses and improve heart health [208]. For instance, a comprehensive study revealed that quercetin displayed remarkable antioxidant activity in human blood plasma, effectively neutralizing harmful free radicals. Furthermore, a study conducted on rats demonstrated that quercetin had anti-inflammatory properties by inhibiting the production of pro-inflammatory molecules, such as tumor necrosis factor-alpha (TNF-α) and interleukin-6 (IL-6), leading to a reduction in the symptoms associated with inflammatory diseases [209,210]. These scientific findings provide compelling evidence supporting the potential health benefits of quercetin and its significance as a valuable dietary component.

Myricetin, on the other hand, is found in foods, such as berries, grapes, and tea, and has comparable antioxidant and anti-inflammatory qualities. Myricetin may have neuroprotective and anticancer properties, according to the research. These adaptable flavonoids are gaining attention in scientific studies as vital components of a balanced diet and possible medicinal possibilities [208]. For instance, the research has revealed that a diet abundant in berries, grapes, and tea, rich sources of myricetin can lower the risk of neurodegenerative diseases, such as Alzheimer’s disease [209]. Moreover, studies have indicated that myricetin can impede the growth of cancer cells and trigger apoptosis, suggesting its potential as a prospective candidate for future cancer treatments [210].

Vendors of natural products in Guyana (known as herbalists colloquially) can use this information on ecotype-specific metabolites; by leveraging the evidence-based research, herbalists can enhance their practices in selecting, preparing, dosing, and utilizing plant materials for medicinal purposes. Scientific insights aid in proper ecotype selection, ensuring authenticity and quality. Herbalists can adopt standardized preparation methods and understand the mechanisms of action of medicinal plants. Moreover, scientific knowledge guides the dosage recommendations, identifies potential interactions, and helps in monitoring treatment effectiveness. By sharing evidence-based information, herbalists can educate patients and contribute to the research, leading to safer and more effective herbal medicine practices.

## 5. Conclusions

The present study aimed to analyze the phytochemical profiles of *D. dentatus* ecotypes using untargeted and semi-targeted plant metabolomics, employing liquid chromatography in conjunction with electrospray ionization mass spectrometry. Our findings highlight the presence of the diverse bioactive compounds in *D. dentatus* ecotypes, including polyphenolic compounds, such as epicatechin methyl gallate, catechin gallate, proanthocyanidin A2 and proanthocyanidin B2 isomers, quercetin 3 glycoside, apigenin, (+)-Catechin, leucocyanidin, anthocyanidin 3-O-beta-D-sambubioside, and resveratrol. These compounds exhibited diverse beneficial biological activities. While this study primarily focused on metabolomics, our future investigations will include antioxidant assays. Antioxidant assays and analyses are integral in comprehending the intricate mechanisms that shield organisms from oxidative stress. These assays thoroughly examine compounds and substances, assessing their potential to counteract the harmful impacts of free radicals and reactive oxygen species. By conducting these assays, the researchers can amass the critical data regarding the efficacy of different antioxidants in mitigating oxidative stress. Using other solvents for metabolite extraction, isolating key therapeutic compounds of interest with confirmation using nuclear magnetic resonance, and testing for bioactivity on select cell lines are potential avenues for future research. This research may provide invaluable insights into the potential interventions and treatments for conditions linked to oxidative stress, encompassing cardiovascular disease, erectile dysfunction, and cancer.

## Figures and Tables

**Table 1:** Tentative identification of selected compounds derived from a combination of classical molecular networking and feature-based molecular networking with fragmentation data grouped according to natural product classification (NP superclass). Tentative identification and/or ionization mode with an asterisk (*) were found in feature-based molecular networking.

Tentative Identification	Molecular Formula	Adduct	Precursor *m*/*z*	MS^2^	NP Superclass
(R)-1-(3,4-dihydroxybenzyl)-1,2,3,4-tetrahydroisoquinoline-6,7-diol	C_16_H_17_NO_4_	M+H	288.12	123.04, 143.05, 149.00, 161.06, 164.0, 225.09	Alkaloids
1-methyl-6,7-dihydroxy-1,2,3,4-tetrahydroisoquinoline (Salsolinol)	C_18_H_24_N_2_O_3_	M+H	180.102	117.07, 137.06, 145.0, 151.07	Alkaloids
Cinchonidine	C_19_H_22_N_2_O	M-H	293.166	59.01, 96.96	Alkaloids
Coniferyl aldehyde	C_10_H_10_O_3_	M+H	179.07	55.02, 91.06, 105.07, 119.05, 123.04, 147.0	Cinnamic acids and derivatives
3-Hydroxy-4-methoxycinnamic acid	C_10_H_10_O_4_	M+H-H_2_O	177.054	89.05, 117.03, 145.0, 149.06	Cinnamic acids and derivatives
6-Methylcoumarin	C_10_H_8_O_2_	M+H	161.06	91.06, 105.07, 115.06, 119.0	Coumarins
(2R,3R,4S,5S,6R)-2-[[2-(3,4-dihydroxyphenyl)-5,7-dihydroxy-3,4-dihydro-2H-chromen-3-yl]oxy]-6-(hydroxymethyl)oxane-3,4,5-triol	C_27_H_30_O_13_	M+H	453.139	85.03, 123.04, 139.0, 163.04, 205.05, 273.08	Flavonoids
Kaempferol *	C_15_H_10_O_6_	M+H	287.055	153.02, 165.02, 213.06, 287.0	Flavonoids
Quercetin	C_15_H_10_O_7_	M+H	303.049	137.02, 151.0, 165.02, 229.05, 257.04	Flavonoids
		M-H	301.035	121.02, 151.0, 179.00	
Maesopsin	C_15_H_12_O_6_	M-H	287.056	57.03, 83.01, 125.0, 151.00, 215.07, 259.06	Flavonoids
(-)-Epicatechin	C_15_H_14_O_6_	M+H	291.086	115.0, 123.04, 139.04	Flavonoids
(+)-Catechin				123.04, 139.0, 147.04, 161.06, 165.05, 179.07, 207.07	Flavonoids
Epigallocatechin	C_15_H_14_O_7_	M+H	307.081	84.08, 111.04, 123.04, 139.0, 151.04	Flavonoids
Gallocatechin		M-H	305.066	109.3, 125.0, 137.02, 161.02, 165.02, 219.07	Flavonoids
Apigetrin [Cosmosiine]	C_21_H_20_O_10_	M+H	433.113	256.73, 271.0	Flavonoids
Astragalin [Kaempferol-3-O-glucoside]	C_21_H_20_O_11_	M+H *	449.108	85.03, 153.02, 287.0	Flavonoids
		M-H	447.091	227.03, 255.03, 284.0	Flavonoids
Kaempferol-4-glucoside		M+H	449.108	85.03, 97.03, 127.04, 287.0	Flavonoids
Isoquercetin *	C_21_H_20_O_12_	M-H	463.088	151.00, 255.03, 271.02, 300.0	Flavonoids
1-(4-hydroxy-3-methoxyphenyl)-6-methoxy-2,3-dimethyl-3,4-dihydro-1H-naphthalene-2,7-diol [6-Methoxyluteolin] *	C_21_H_22_O_6_	M+H-H_2_O	327.158	137.06, 151.0, 171.08, 203.11	Flavonoids
Phlorizin [3-(beta-D-glucopyranosyloxy)-2-methyl-4H-Pyran-4-one]	C_21_H_24_O_10_	M-H	435.13	125.02, 167.0, 179.03, 273.08	Flavonoids
		M+H *	289.092	85.03, 127.0	Flavonoids
(-)-Catechin gallate	C_22_H_18_O_10_	M+H	443.096	123.04, 139.0, 153.02, 273.07	Flavonoids
Epicatechin gallate		M-H	441.08	109.03, 125.02, 169.0, 193.01, 245.08, 289.07	Flavonoids
		M+H *	443.097	139.0, 153.02, 165.05, 273.07, 291.09	Flavonoids
[6-[5,7-dihydroxy-2-(4-hydroxyphenyl)-4-oxochromen-3-yl]oxy-3,4,5-trihydroxyoxan-2-yl]methyl 3,4,5-trihydroxybenzoate	C_28_H_24_O_15_	M-H	599.105	125.02, 151.00, 169.0, 284.03, 285.04, 313.06	Flavonoids
Procyanidin B1	C_30_H_26_O_12_	M-H	577.135	109.0, 125.0, 161.03, 203.07, 245.08, 289.07, 407.08	Flavonoids
Procyanidin B2		M+H	579.15	127.0, 139.04, 191.03, 233.04, 247.06, 271.06, 287.06, 409.09	Flavonoids
Isatin	C_8_H_5_NO_2_	M+H	148.04	92.05, 120.0	Indoles and derivatives
[3,4,5-trihydroxy-6-(3,4,5-trihydroxybenzoyl)oxyoxan-2-yl]methyl3,4,5-trihydroxybenzoate	C_20_H_20_O_14_	M-H	483.078	125.02, 151.01, 169.0, 211.02, 271.04, 313.07	Phenolic acids
3,4-Dihydroxy-5-[(6-O-{[4-(2-hydroxy-2-propanyl)-1-cyclohexen-1-yl]carbonyl}-beta-D-glucopyranosyl)oxy]benzoic acid	C_23_H_30_O_12_	M-H	497.166	125.02, 169.0, 313.06, 331.07	Phenolic acids
Gallic acid	C_7_H_6_O_5_	M+H-H_2_O	153.018	79.02, 97.03, 107.0, 125.02	Phenolic acids
		M-H *	169.014	81.03, 97.03, 125.0, 169.01	
4-Allyl-2,6-dimethoxyphenol (Eugenol) *	C_10_H_12_O_3_	M+H	195.102	107.0, 135.08, 154.06, 163.07, 195.10	Phenolic compounds
Genipin *	C_11_H_14_O_5_	M+H-H_2_O	209.081	121.0, 149.06, 177.06, 181.05, 209.08	Phenolic compounds
Coumaroyl quinic acid *	C_16_H_18_O_9_	M-H	337.092	93.03, 163.04, 173.04, 191.0	Phenolic compounds
Protocatechuic acid *	C_7_H_6_O_4_	M-H	137.024	93.03, 108.02, 109.03, 137.0	Phenolic compounds
p-Coumaric acid *	C_9_H_8_O_3_	M+H-H_2_O	147.044	91.05, 119.0, 123.96, 147.04	Phenolic compounds
Methyl gallate *	C_8_H_8_O_5_	M+H	185.045	113.01, 126.03, 153.02, 185.0	Phenolic compounds (gallotannins)
Cuminyl alcohol *	C_10_H_12_O_2_	M+H-H_2_O	133.101	91.05, 105.07, 118.08, 133.1	Phenolic compounds (phenylpropanoids)
Alpha-Pinene *	C_10_H_16_	M+H	137.13	67.05, 79.05, 81.07, 95.09, 109.10, 137.10	Terpenoids
1S,2S,6R,7R,9R)-6-methyl-10,12-dioxatricyclo [7.2.1.0<2,7>]dodec-4-en-8-one [Ascaridole] *	C_10_H_16_O_2_	M+H	195.102	95.05, 123.08, 149.06, 167.07	Terpenoids
(1R)-(-)-Nopol	C_11_H_18_O	M+H-H_2_O	149.132	65.04, 81.07, 93.07, 107.09, 121.0	Terpenoids
3-(beta-D-glucopyranosyloxy)-2-methyl-4H-Pyran-4-one	C_13_H_20_O_8_	M+H	289.092	97.03, 127.0	Terpenoids
4-(2,6,6-trimethyl-2-cyclohexen-1-yl)- 2-Butanone	C_15_H_22_O	M+H	177.164	79.06, 81.07, 93.07, 95.09, 107.09, 1121.1, 149.06	Terpenoids
Farnesol *	C_15_H_26_O	M+H	223.205	69.07, 95.09, 83.09, 109.10, 121.10, 135.12, 205.20, 223.21	Terpenoids
Betulinic acid	C_30_H_48_O_3_	M+H-H_2_O	439.357	57.07, 81.07, 95.09, 109.10, 123.12, 137.13, 189.16, 241.19, 255.21	Terpenoids
Betulonic acid	C_30_H_48_O_4_	M+H-H_2_O	437.341	69.07, 95.09, 107.09, 121.10, 135.12, 189.16, 203.18, 215.18, 241.19, 255.21, 391.33	Terpenoids
Sumaresinolic acid	C_30_H_48_O_4_	M+H-H_2_O	455.352	57.07, 81.07, 95.09, 109.10, 137.13, 189.16, 203.18, 391.33, 409.35	Terpenoids
Betulin	C_30_H_50_O_2_	M+H-H_2_O	425.378	67.06, 81.07, 95.09, 109.10, 123.12, 137.13, 189.16, 201.16,215.18, 227.18, 255.21, 269.23	Terpenoids

**Table 2:** Tentative identification of compounds resulting from data analysis using MS DIAL grouped according to natural product classification (NP superclass). Tentative identifications that were not seen in the GNPS workflow are denoted by an asterisk (*).

Tentative Identification	Molecular Formula	Adduct	Precursor *m*/*z*	MS^2^	NP Superclass
Epicatechin gallate	C_22_H_18_O_10_	M+H	443.0972	139.039; 153.0184; 165.0548	Flavonoids (catechin gallates)
Quercetin	C_15_H_10_O_7_	M+H	303.0499	137.0240, 229.0488	Flavonoids (flavonols)
Methylophiopogonanone A *	C_19_H_18_O_6_	M+Na	365.1052	185.0422; 203.0528; 365.1057	Flavonoids (homoisoflavanones)
Emodin *	C_15_H_10_O_5_	M-H	269.0452	201.0566; 241.0503; 269.0546	Phenolic compounds (hydroxyanthraquinones)
Catechol	C_6_H_6_O_2_	M-H	109.0293	81.0345, 91.0189, 108.0217, 109.0294	Phenolic compounds
3-Coumaric acid *	C_9_H_8_O_3_	M-H	163.0402	119.0499, 163.0034	Phenolic compounds (hydroxycinnamic acids)
p-Coumaric acid	C_9_H_8_O_3_	M+H	165.0546	91.0540, 95.0858, 119.0493, 147.0442	Phenolic compounds (hydroxycinnamic acids)
Caffeic acid *	C_9_H_8_O_4_	M-H-CO_2_	135.0451	107.0499, 135.0450	Phenolic compounds (hydroxycinnamic acids)
2-[(2S,4aR,8aS)-2-hydroxy-4a-methyl-8-methylidene-3,4,5,6,7,8a-hexahydro-1H-naphthalen-2-yl]prop-2-enoic acid *	C_15_H_22_O_3_	M-H	249.1495	205.1594, 249.1494	Terpenoids (eudesmane, isoeudesmane, or cyclic terpenoids)
Saikikogenin D *	C_30_H_48_O_4_	M+H	473.3625	473.3460 (only MS^1^ available)	Terpenoids (triterpenoids)

## Data Availability

All relevant data generated or analyzed during this study are included in this published article and Supplementary Materials. Other raw data are available upon request.

## Supplementary Materials

The following supporting information can be downloaded at: https://www.mdpi.com/article/10.3390/metabo13101050/s1, Table S1: Tentative metabolite identification of compounds that are biofingerprints of interests found in both *D. dentatus* red and white ecotypes. SIRIUS was used as an in silico fragmentation tool for metabolite annotation. Tentative identification of compounds with fragments are at level 3 using SIRIUS, otherwise at level 4 metabolite annotation; Table S2: Tentative metabolite identification of compounds that are biofingerprints of interests unique to *D. dentatus* red ecotype. SIRIUS was used as an in silico fragmentation tool for metabolite annotation. Tentative identification of compounds with fragments are at level 3 using SIRIUS, otherwise at level 4 metabolite annotation; Table S3: Tentative metabolite identification of compounds that are biofingerprints of interests unique to *D. dentatus* white ecotype. SIRIUS was used as an in silico fragmentation tool for metabolite annotation. Tentative identification of compounds with fragments are at level 3 using SIRIUS, otherwise at level 4 metabolite annotation; Table S4: Tentative metabolite identification of flavonoid biofingerprints of interests found in *D. dentatus* red and white ecotypes. SIRIUS was used as an in silico fragmentation tool for metabolite annotation. Tentative identification of compounds with fragments are at level 3 using SIRIUS, otherwise at level 4 metabolite annotation; Table S5: Tentative metabolite identification of alkaloid biofingerprints of interests found in *D. dentatus* red and white ecotypes. SIRIUS 5.8.3 was used as an in silico fragmentation tool for metabolite annotation. Tentative identification of compounds with fragments are at level 3 using SIRIUS, otherwise at level 4 metabolite annotation; Table S6: Tentative metabolite identification of terpenoid biofingerprints found in *D. dentatus* red and white ecotypes. SIRIUS 5.8.3 was used as an in silico fragmentation tool for metabolite annotation. Tentative identification of compounds with fragments are at level 3 using SIRIUS, otherwise at level 4 metabolite annotation; Table S7: Tentative identification of other compounds outside of alkaloids, terpenoids and flavonoids found in both *D. dentatus* red and white ecotypes. SIRIUS was used as an in silico fragmentation tool for metabolite annotation. Tentative identification of compounds with fragments are at level 3 using SIRIUS, otherwise at level 4 metabolite annotation; Table S8: Pathway impacts of biochemical pathways in *Doliocarpus dentatus* white ecotype as a result of using the mummichog algorithm used in Pathway analysis module in MetaboAnalyst 5.0; Table S9: Biochemical pathway impacts observed in *Doliocarpus dentatus* red ecotype as a result of using the mummichog algorithm used in Pathway analysis module in MetaboAnalyst 5.0; Figure S1: VIP scores for PLS-DA showing features responsible for divergence from PCA analysis. (A) represents data in positive ionization mode and (B) negative ionization mode; Figure S2: Bar chart showing up-or-downregulated select putative compounds at level 4 metabolite annotation. Metabolite annotation was done via MetaboAnalyst 5.0, MetaboQuest and linked to KEGG. Xcalibur Software (v.4.1) was used to confirm masses and peak quality in sample replicates; Figure S3: Select terpenoid biomarkers showing molecular structure and formulae, chromatogram peak region found, and integrated peak areas. For integrated peak areas, red and green denote red and white Capadulla respectively.; Figure S4: Select alkaloid biomarkers showing molecular structure and formulae, chromatogram peak region found, and integrated peak areas. For integrated peak areas, red and green denote red and white Capadulla respectively; Figure S5: Select phenolic biomarkers showing molecular structure and formulae, chromatogram peak region found, and integrated peak areas. For integrated peak areas, red and green denote red and white Capadulla respectively; Figure S6A: Select level 2 tentative identification of flavonoid compounds in positive ionization mode using MS-DIAL 5.1; Figure S6B: Select level 2 tentative identification of flavonoid compounds in positive ionization mode using MS-DIAL 5.1; Figure S7: Merged polarity network analysis of classical molecular networking for *D. dentatus* ecotypes using positive and negative ionization modes; Figure S8: Merged polarity network analysis of feature-based molecular networking for *D. dentatus* ecotypes using positive and negative ionization modes. Additional excel tables and tsv files for SIRIUS outputs and other annotations.
