# Supplementary material for: Unlocking Potentially Therapeutic Phytochemicals in Capadulla (Doliocarpus dentatus) from Guyana Using Untargeted Mass Spectrometry-Based Metabolomics
Source: Metabolites. 2023 Oct 3;13(10):1050. doi: 10.3390/metabo13101050 (PMC10608729; doi:10.3390/metabo13101050)
Supplement: Supplementary file 1 [file metabolites-13-01050-s001.zip › Supplemental Information Capadulla Dolio September 14 2023.pdf]

Supplemental Information

Unlocking potentially therapeutic phytochemicals in Capadulla (*Doliocarpus dentatus*) from Guyana using untargeted mass spectrometry-based metabolomics

Ewart Smith[a], Ainsely Lewis[b], RJ Neil Emery[b], and Suresh S Narine[c]

[a]Environmental and Life Sciences Graduate Program, Trent University, Peterborough, ON, Canada

[b]Department of Biology, Trent University, Peterborough, ON, Canada

[c]Trent Centre for Biomaterials Research, Trent University, Peterborough, ON, Canada

Corresponding Authors: ewartsmith@trentu.ca , ainselylewis@trentu.ca

1. Method

1.1 XCMS Online Settings for data alignment and annotation

XCMS Online default values were used for analysis. Briefly, pairwise comparisons were used with UPLC/Orbitrap parameter 3110. For feature detection, the centWave option was used with 5 ppm error, and a minimum and maximum peak width of 5 and 20 respectively. The minimum mz difference (mzdiff) was 0.01 with a signal/noise threshold of 4, and 1 was used for the integration method. For retention time correction, the Obiwarp method was used. For alignment, the mz width used (mzwid) was 0.025, with a bandwidth (bw) of 5, and a minimum fraction of samples (minfrac) of 0.5. the minimum number of samples (minsamp) was 1 with a maximum number of groups of 100.

For statistical analyses, the unpaired parametric t test (Welch t test) was used with a p-value threshold of 0.01 for highly significant features, and a foldchange threshold of 1.5. p-value threshold of significant features was 0.05. For annotation, both isotopes and adducts were searched for at a 5 ppm error with an m/z absolute error of 0.015.

1.2 Using SIRIUS 5.8.3 for metabolite annotation

1.2.1 Positive ionization mode settings

config --IsotopeSettings.filter=true --FormulaSearchDB=BIO,METACYC,CHEBI,COCONUT,ECOCYCMINE,GNPS,HMDB,HSDB,KEGG,KEGGMINE,KNAPSACK,MACONDA,MESH,NORMAN,UNDP,PLANTCYC,PUBCHEM,PUBMED,YMDB,YMDBMINE,ZINCBIO --Timeout.secondsPerTree=0 --FormulaSettings.enforced=HCNOP --Timeout.secondsPerInstance=0 --AdductSettings.detectable=[[M+H]+,[M+H3N+H]+,[M-H4O2+H]+,[M-H2O+H]+] --UseHeuristic.mzToUseHeuristicOnly=650 --AlgorithmProfile=orbitrap --IsotopeMs2Settings=IGNORE --MS2MassDeviation.allowedMassDeviation=10.0ppm --NumberOfCandidatesPerIon=1 --UseHeuristic.mzToUseHeuristic=300 --FormulaSettings.detectable=Cl,S --NumberOfCandidates=10 --ZodiacNumberOfConsideredCandidatesAt300Mz=10 --ZodiacRunInTwoSteps=true --ZodiacEdgeFilterThresholds.minLocalConnections=10 --ZodiacEdgeFilterThresholds.thresholdFilter=0.95 --ZodiacEpochs.burnInPeriod=2000 --ZodiacEpochs.numberOfMarkovChains=10 --ZodiacNumberOfConsideredCandidatesAt800Mz=50 --ZodiacEpochs.iterations=20000 --AdductSettings.enforced=, --AdductSettings.fallback=[[M+H]+,[M-H2O+H]+] --FormulaResultThreshold=true --InjectElGordoCompounds=true --StructureSearchDB=BIO,METACYC,CHEBI,COCONUT,ECOCYCMINE,GNPS,HMDB,HSDB,KEGG,KEGGMINE,KNAPSACK,MACONDA,MESH,NORMAN,UNDP,PLANTCYC,PUBCHEM,PUBMED,YMDB,YMDBMINE,ZINCBIO --RecomputeResults=false formula zodiac fingerprint structure canopus

1.2.2 Negative ionization mode settings

config --IsotopeSettings.filter=true --FormulaSearchDB=BIO,METACYC,CHEBI,COCONUT,ECOCYCMINE,GNPS,HMDB,HSDB,KEGG,KEGGMINE,KNAPSACK,MACONDA,MESH,NORMAN,UNDP,PLANTCYC,PUBCHEM,PUBMED,YMDB,YMDBMINE,ZINCBIO --Timeout.secondsPerTree=0 --FormulaSettings.enforced=HCNOP --Timeout.secondsPerInstance=0 --AdductSettings.detectable=[[M-H]-,[M-H2O-H]-] --UseHeuristic.mzToUseHeuristicOnly=650 --AlgorithmProfile=orbitrap --IsotopeMs2Settings=IGNORE --MS2MassDeviation.allowedMassDeviation=10.0ppm --NumberOfCandidatesPerIon=1 --UseHeuristic.mzToUseHeuristic=300 --FormulaSettings.detectable=B,Cl,Br,Se,S --NumberOfCandidates=10 --ZodiacNumberOfConsideredCandidatesAt300Mz=10 --ZodiacRunInTwoSteps=true --ZodiacEdgeFilterThresholds.minLocalConnections=10 --ZodiacEdgeFilterThresholds.thresholdFilter=0.95 --ZodiacEpochs.burnInPeriod=2000 --ZodiacEpochs.numberOfMarkovChains=10 --ZodiacNumberOfConsideredCandidatesAt800Mz=50 --ZodiacEpochs.iterations=20000 --AdductSettings.enforced=, --AdductSettings.fallback=[[M-H]-,[M-H2O-H]-] --FormulaResultThreshold=true --InjectElGordoCompounds=true --StructureSearchDB=BIO,METACYC,CHEBI,COCONUT,ECOCYCMINE,GNPS,HMDB,HSDB,KEGG,KEGGMINE,K

```
NAPSACK,MACONDA,MESH,NORMAN,UNDP,PLANTCYC,PUBCHEM,PUBMED,YMDB,YMDBMINE,ZINCBIO
--RecomputeResults=false formula zodiac fingerprint structure canopus
```

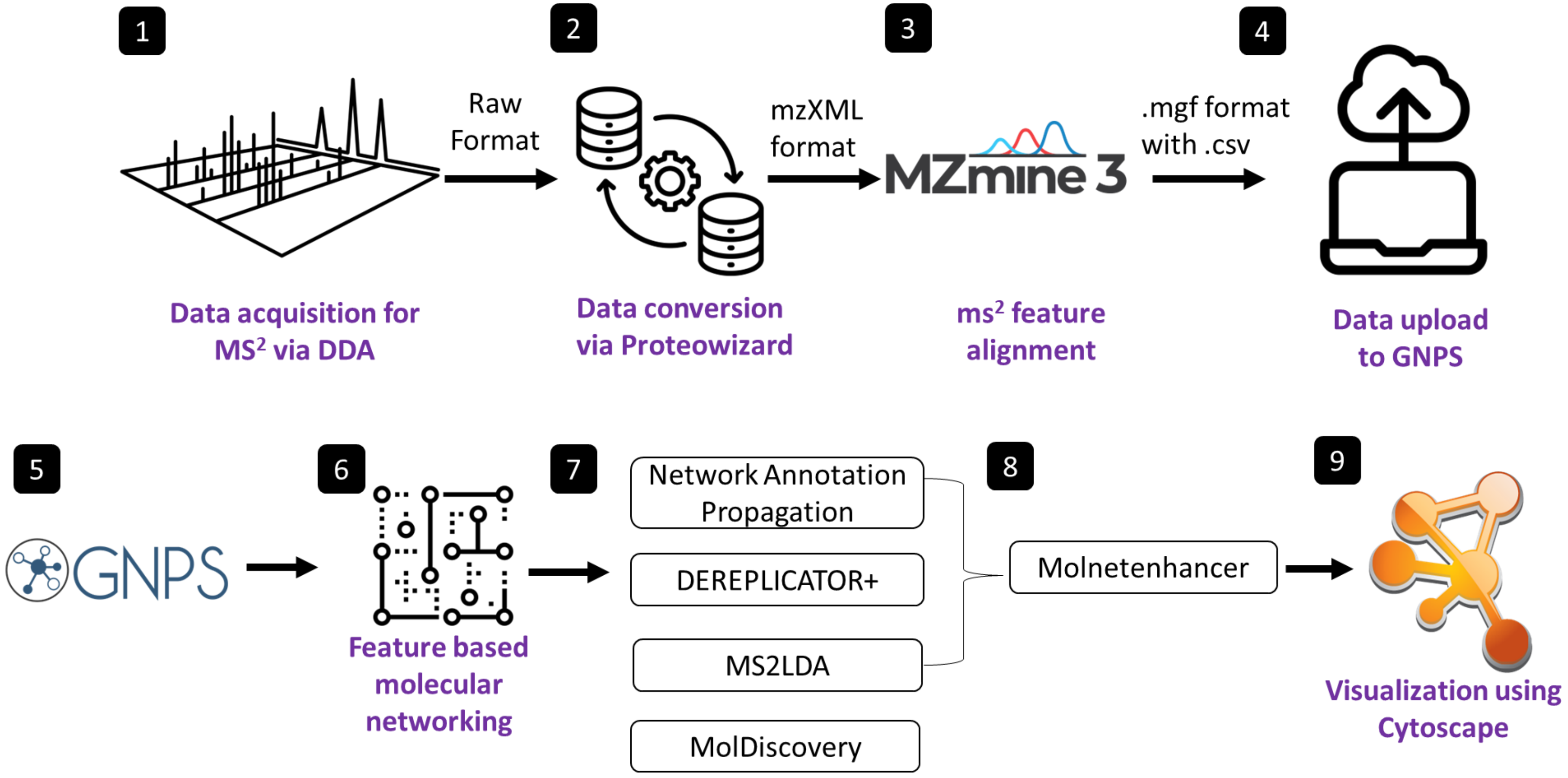

Figure S1: (A) Workflow of tandem mass spectrometry processing for preparation using feature based molecular networking via GNPS.

Suplemental Table S1: Tentative metabolite identification of compounds that are biofingerprints of interests found in both *D. dentatus* red and white ecotypes. SIRIUS was used as an in silico fragmentation tool for metabolite annotation. Tentative identification of compounds with fragments are at level 3 using SIRIUS, otherwise at level 4 metabolite annotation.

Tentative Identification	Molecular formula	Adduct	Exact mass	Theoretical mass	Experimental mass	Mass error (ppm)	t_R (min)	MS^2	Annotation Level
Resveratrol	$C_{14}H_{12}O_3$	M+H	228.0786	228.0786	228.0773	5.89	4.0	-	4
Quercetin	$C_{15}H_{10}O_7$	M-H	302.0427	301.0354	301.0356	-0.74	4.0	215.0697,149.0241,243.0668	3
Epicatechin 3-O-(3'-O-Methyl gallate)	$C_{23}H_{20}O_{10}$	M+H	456.1056	457.1129	457.1115	3.06	4.6	167.0346,139.0398,123.0440	3
(+)-Catechin	$C_{15}H_{14}O_6$	M+H	290.079	291.0863	291.0851	4.17	4.4	245.0823,203.0717,109.0292	2
Apigenin	$C_{15}H_{10}O_5$	M+H	270.0528	271.0601	271.0591	3.69		215.0698,194.0242,243.0668	3
Proanthocyanidin B2	$C_{30}H_{26}O_{12}$	M+H	578.1424	579.1497	579.1476	3.63	4.5	127.0387, 163.0395,247.0615	3
Leucocyanidin	$C_{15}H_{14}O_7$	M+H	306.074	307.0812	307.0801	3.68	4.1	-	4
Luteolin	$C_{15}H_{10}O_6$	M+H	286.0477	287.0550	287.0541	3.19	4.9	295.0606,167.0695,153.0191	3
Naringenin	$C_{15}H_{12}O_5$	M+H	272.0685	273.0758	273.0746	4.21	5.0		4
Anthocyanidin 3-O-beta-D-sambubioside	$C_{26}H_{29}O_{15}$	M+H	581.1506	582.1579	582.1564	2.61	4.1	-	4
Catechin 3-O-Beta-D-Glucopyranoside	$C_{21}H_{24}O_{11}$	M+H	452.1319	453.1391	453.1371	4.41	4.1	289.0710,245.0823,271.0621	4
Taxifolin	$C_{15}H_{12}O_7$	M+H	304.0583	305.0656	305.0645	3.54	4.3	217.0486, 201.0564,187.0764	3
Quercetin 3-glucoside	$C_{21}H_{20}O_{12}$	M+H	464.0955	465.1028	465.102	1.62	4	-	4
Lyoniside	$C_{27}H_{36}O_{12}$	M+H	552.2207	551.2134	551.2095	7.07	4.3	-	4
Proanthocyanidin A2	$C_{30}H_{24}O_{12}$	M+H	578.1424	579.1497	579.1476	3.63	4.2	245.0449,287.0565,275.0569	3
Chelidonine	$C_{20}H_{19}NO_5$	M+H	353.1263	354.1336	354.1349	-3.67	5.5	-	4
Trigonelline	$C_7H_7NO_2$	M+H	137.0477	138.0550	138.0544	4.02	0.8	-	4
Kyanmethin	$C_6H_9N_3$	M+H	123.0796	124.0869	124.0865	3.22	7.1	107.0606,810703,79.0545	3
4-Methylpelletierine	$C_{10}H_{13}NO_4$	M+H	211.0845	212.0917	212.0908	4.24	7.1	194.0811,166.0866,109.0287	3
Salsolinol	$C_9H_{11}NO_2$	M+H	165.0790	166.0863	166.0857	3.34	3.9	178.0498,132.0448,105.0338	3
Nicotinamide	$C_6H_6N_{2O}$	M+H	122.0480	123.0553	123.0548	3.98	0.7	163.0753,145.0652,117.0702	3
Kynurenate	$C_{10}H_7NO_3$	M+H	189.0426	190.0499	190.0491	4.21	4.1	162.0549,144.0438,116.0499	3
Raphanatin	$C_{16}H_{23}N_5O_6$	M+H	381.1648	382.1721	382.1704	4.47	3.8	139.0627,202.1009,145.0509	3
Perillic acid	$C_{10}H_{14}O_2$	M+H	166.0994	167.1067	167.1063	2.13	4.6	214.1010,187.1130,149.0605	3

(+)-Pulegone	$C_{10}H_{16}O$	M+H	152.1201	153.1274	153.1270	2.61	3.7	107.0857,97.0650,95.0860	3
Xerantholide	$C_{15}H_{18}O_3$	M+H	246.1256	247.1329	247.1322	2.83	4.7	214.1010,187.1130,149.0605	3
Kaurane -17,18-dioic acid	$C_{20}H_{30}O_4$	M+H	334.2144	335.2217	335.2204	3.89	4.6	153.0189, 171.0295, 139.0397	3
Betulonic Acid	$C_{30}H_{46}O_3$	M+H	454.3447	455.3520	455.3502	3.89	7	137.1332,95.0861,203.1792	3
Lupenone	$C_{30}H_{48}O$	M-H	424.3705	425.3778	425.3762	3.74	6.9	95.0860,81.0700,137.1332	3
Geniposidic acid	$C_{16}H_{22}O_{10}$	M-H	374.1213	373.1140	373.1115	6.70	3.7	193.0504,343.1048,181.0498	3
Buddlejoside B	$C_{22}H_{26}O_{12}$	M-H	482.1424	481.1351	481.1312	8.21	4.2	169.0138,3130573,151.0042	3
Diospyric Acid B	$C_{30}H_{46}O_6$	M-H	502.3294	501.3222	501.3203	3.72	5.1	439.3207,457.3303,469.3316	3
Glucosol	$C_{30}H_{48}O_4$	M-H	472.3553	471.3480	471.3461	4.00	5.9	427.3587,469.3331,423.3260	3
(-)-Lariciresinol	$C_{20}H_{24}O_6$	M+H	360.1573	361.1646	361.1642	1.01	4.4	-	4
(-)-Pinoresinol	$C_{20}H_{22}O_6$	M+H	358.1416	359.1489	359.1475	3.94	4.7	-	4
Palmatoside G	$C_{25}H_{32}O_{10}$	M+H	492.1995	493.2068	493.2043	5.12	4	-	4
Gallate	$C_7H_6O_5$	M-H	170.0215	169.0142	169.0134	5.01	0.5	125.0245,97.0290,79.0186	3
2-hydroxy-5-[(3,4,5-trihydroxyoxan-2-yl) oxy]benzoic acid	$C_{12}H_{14}O_8$	M-H	286.0689	285.0616	285.0599	5.96	3.9	152.0106,108.0213,109.0291	3
Lyoniside	$C_{27}H_{36}O_{12}$	M-H-	552.2207	551.2134	551.2095	7.07	4.3	373.1282,404.1492,59.0137	3
Gentisic acid	$C_7H_6O_4$	M-H-	154.0266	153.0193	153.0183	6.74	3	109.0292,110.0010,137.9956	3

Supplemental Table S2: Tentative metabolite identification of compounds that are biofingerprints of interests unique to *D. dentatus* red ecotype. SIRIUS was used as an in silico fragmentation tool for metabolite annotation. Tentative identification of compounds with fragments are at level 3 using SIRIUS, otherwise at level 4 metabolite annotation.

Tentative Identification	Molecular formula	Ionization Mode	Exact mass	Theoretical mass	Experimental mass	Mass error (ppm)	t_R (min)	MS^2	Annotation Level
(-)-Epigallocatechin	$C_{15}H_{14}O_7$	M+H	306.0740	307.0812	307.0812	4.33	4.2	-	4
(+)-alpha-Barbatene	$C_{15}H_{24}$	M+H	204.1878	205.1951	205.1943	3.79	5.2	-	4
(+)-Pulegone	$C_{10}H_{16}O$	M+H	152.1201	153.1274	153.1270	2.61	3.7	107.0857,97.0650,95.0860	3
3-alpha(S)-Strictosidine	$C_{27}H_{34}N_2O_9$	M+H	530.2264	531.2337	531.2332	0.95	6	-	4
Afzelin	$C_{21}H_{20}O_{10}$	M+H	432.1056	433.1129	433.1117	2.82	4.5	-	4
alpha-Amyrin	$C_{30}H_{50}O$	M+H	426.3862	427.3934	427.3923	2.67	6.5	-	4
Anthocyanidin 3-O-beta-D-sambubioside	$C_{26}H_{29}O_{15}$	M+H	581.1506	582.1579	582.1564	2.61	4.1	-	4
Cyanidin 3-O-beta-D-sambubioside	$C_{27}H_{31}O_{14}+$	M+H	579.1700	580.1787	580.1841	-9.38	4.6	-	4
Coniferyl Alcohol	$C_{10}H_{12}O_3$	M+H	180.0786	181.0859	181.0853	3.43	5.1	-	4
Gibberellin A_1	$C_{19}H_{24}O_6$	M+H	348.1573	349.1646	349.1671	-7.26	4.0	-	4
Gibberellin A_{19}	$C_{20}H_{26}O_6$	M+H	362.1729	363.1802	363.1788	3.90	5.0	-	4
Katuranin	$C_{15}H_{12}O_6$	M+H	288.0634	287.0561	287.0541	7.00	4.3	215.0697,149.0241,243.0668	3
Leucocyanidin	$C_{15}H_{14}O_7$	M+H	306.074	307.0812	307.0801	3.68	4.1	-	4
Luteolin	$C_{15}H_{10}O_6$	M+H	286.0477	287.0550	287.0541	3.19	4.9	-	4
Naringenin	$C_{15}H_{12}O_5$	M+H	272.0685	273.0758	273.0746	4.21	5.0	-	4
Pheophorbide a	$C_{35}H_{36}N_4O_5$	M+H	592.2686	593.2758	593.2781	-3.80	5.1	-	4
Quercitrin	$C_{21}H_{20}O_{11}$	M+H	448.1006	449.1078	449.1069	2.00	4.5	278.0559,61.0287,71.0494	3

Supplemental Table S3: Tentative metabolite identification of compounds that are biofingerprints of interests unique to *D. dentatus* white ecotype. SIRIUS was used as an in silico fragmentation tool for metabolite annotation. Tentative identification of compounds with fragments are at level 3 using SIRIUS, otherwise at level 4 metabolite annotation.

Tentative Identification	Molecular formula	Ionization Mode	Exact mass	Theoretical mass	Experimental mass	Mass error (ppm)	t_R (min)	MS^2	Annotation Level
Nicotinic acid	$C_6H_6N_2O$	M+H	122.0480	123.0553	123.0548	3.98	4.7	-	4
Perillyl aldehyde	$C_{10}H_{14}O$	M+H	150.1045	151.1117	151.1113	2.92	4.1	-	4
5-Hydroxyindoleacetaldehyde	$C_{10}H_9NO_2$	M+H	175.0633	176.0706	176.0702	2.30	4.5	-	4
ent-Kaurene	$C_{20}H_{32}$	M+H	272.2504	273.2577	273.2567	3.58	6	-	4
Myricetin	$C_{15}H_{10}O_8$	M+H	318.0376	319.0448	319.0442	2.02	4.1	-	4
Gibberellin A_{51}-catabolite	$C_{19}H_{22}O_5$	M+H	330.1467	331.1540	331.1534	1.81	4.5	-	4
L-Dopa	$C_9H_{11}NO_4$	M+H	197.0688	198.0761	198.0754	3.46	3.8	-	4
Gibberellin A_{20}	$C_{19}H_{24}O_5$	M+H	332.1624	333.1697	333.168	4.95	5.02	-	4
Gibberellin A_4	$C_{20}H_{28}O_6$	M+H	364.1886	364.1886	364.1880	1.62	4.5	-	4
Gibberellin A_{51}	$C_{19}H_{22}O_5$	M+H	330.1467	331.1540	331.1534	1.81	4.5	-	4
Quercetin	$C_{15}H_{10}O_7$	M-H	302.0427	301.0354	301.0356	-0.74	4.01	163.0396,147.0444,137.0237	3

Supplemental Table S4: Tentative metabolite identification of flavonoid biofingerprints of interests found in *D. dentatus* red and white ecotypes. SIRIUS was used as an in silico fragmentation tool for metabolite annotation. Tentative identification of compounds with fragments are at level 3 using SIRIUS, otherwise at level 4 metabolite annotation.

Tentative Identification	Molecular formula	Ionization Mode	Exact mass	Theoretical mass	Experimental mass	Mass error (ppm)	t_R (min)	MS^2	Annotation Level
5-Hydroxyconiferyl alcohol	$C_{10}H_{12}O_4$	M+H	196.0736	197.081	197.0802	3.04	4.2	-	4
Apigenin	$C_{15}H_{10}O_5$	M+H	270.0528	271.0601	271.0591	3.69	4.5	215.0698,194.0242,243.0668	3
Luteolin	$C_{15}H_{10}O_6$	M+H	286.0477	287.0550	287.0541	3.19	4.9	231.0667,213.0561,153.0191	3
Myricetin	$C_{15}H_{10}O_8$	M+H	318.0376	319.0448	319.0442	2.02	4.1	-	4
3,9-Dihydroxypterocarpan	$C_{15}H_{12}O_4$	M+H	256.0736	257.0808	257.0802	2.47	4.74	-	4
(+-)-Dalbergioidin	$C_{15}H_{12}O_6$	M+H	288.0634	289.0707	289.0697	3.34	4.2	-	4
Taxifolin	$C_{15}H_{12}O_7$	M+H	304.0583	305.0656	305.0645	3.54	4.3	217.0486, 01.0564,187.0764	3
Dihydromyricetin	$C_{15}H_{12}O_8$	M+H	320.0532	321.0605	321.0593	3.72	4.1	-	4
(+)-Catechin	$C_{15}H_{14}O_6$	M+H	290.079	291.0863	291.0851	4.17	4.4	139.0398,177.0558,123.0439	2
Leucodelphinidin	$C_{15}H_{14}O_8$	M+H	322.0689	323.0761	323.0750	3.54	4.5	-	4
Quercitrin	$C_{21}H_{20}O_{11}$	M+H	448.1006	449.1078	449.1069	2.00	4.5	278.0559,61.0287,71.0494	3
Quercetin 3-glucoside	$C_{21}H_{20}O_{12}$	M+H	464.0955	465.1028	465.102	1.62	4.4	-	4
Cristacarpin	$C_{21}H_{22}O_5$	M+H	354.1467	355.1540	355.1526	3.94	5.04	-	4
Catechin 3-O-Beta-D-Glucopyranoside	$C_{21}H_{24}O_{11}$	M+H	452.1319	453.1391	453.1371	4.41	4.09	139.0399,123.0440,163.0398	3
Fisetin	$C_{22}H_{16}O_8$	M+H	408.0845	409.0918	409.0902	3.91	4.5	257.0437,285.0414,229.0512	3
Epicatechin 3-O-(3'-O-Methylgallate)	$C_{23}H_{20}O_{10}$	M+H	456.1056	457.1129	457.1115	3.06	4.6	167.0346,139.0398,123.0440	3
Anthocyanidin 3-O-beta-D-sambubioside	$C_{26}H_{29}O_{15}$	M+H	581.1506	582.1579	582.1564	2.61	4.1	-	4
Proanthocyanidin A2	$C_{30}H_{24}O_{12}$	M+H	576.1268	577.1341	577.1324	2.94	4.5	245.0449,287.0565,275.0569	3
Gambiriin C	$C_{30}H_{26}O_{11}$	M+H	562.1475	563.1548	563.1536	2.11	4.5	139.0399,147.0445,285.0779	3
Proanthocyanidin B2	$C_{30}H_{26}O_{12}$	M+H	578.1424	579.1497	579.1476	3.63	4.2	127.0387, 63.0395,247.0615	4

Quercetin	$C_{15}H_{10}O_7$	M-H	302.0427	301.0354	301.0356	-0.74	4.06	163.0396,147.0444,137.0237	3
Katuranin	$C_{15}H_{12}O_6$	M-H	288.0634	287.0561	287.0541	7.01	4.3	215.0697,149.0241,243.0668	3
Resveratrol	$C_{14}H_{12}O_3$	M+H	228.0786	228.0786	228.0773	5.89	4.7	-	4
Distylin	$C_{15}H_{12}O_7$	M-H	304.0583	303.0510	303.049	6.69	4.27	125.0243,235.0618,258.0396	3
(+)-Catechin	$C_{15}H_{14}O_6$	M-H	290.079	289.0718	289.0695	7.82	4.27	245.0823,203.0717,109.0292	2
Astragalin	$C_{21}H_{20}O_{11}$	M-H	448.1006	447.0933	447.0966	-7.42	4.5	285.0396,284.0330,257.0467	3
Engeletin	$C_{21}H_{22}O_{10}$	M-H	434.1213	433.1140	433.1117	5.36	4.61	269.0446,180.0055,152.0107	3
Astilbin	$C_{21}H_{22}O_{11}$	M-H	450.1162	449.1089	449.1089	0.08	5	241.0503,169.0662,199.0403	3
Quercetin 7-(6"-Acetylglucoside)	$C_{23}H_{22}O_{13}$	M-H	506.1060	505.0988	505.0989	-0.27	4.13	233.0810,183.0295,215.0709	3
Endotelon	$C_{30}H_{26}O_{12}$	M-H	578.1424	577.1351	577.1324	4.763	4.23	125.0243,245.0823,283.0235	3

Supplemental Table S5: Tentative metabolite identification of alkaloid biofingerprints of interests found in *D. dentatus* red and white ecotypes. SIRIUS 5.8.3 was used as an in silico fragmentation tool for metabolite annotation. Tentative identification of compounds with fragments are at level 3 using SIRIUS, otherwise at level 4 metabolite annotation.

Tentative Identification	Molecular formula	Ionization Mode	Exact mass	Theoretical mass	Experimental mass	Mass error (ppm)	t_R (min)	MS^2	Annotation Level
Tyramine	$C_8H_{11}NO$	M+H	137.0841	138.0913	138.0908	3.91	6.9	-	4
Thiazole	C_6H_9NOS	M+H	143.0405	144.0478	144.0473	3.20	1.6	98.0605,84.0811,84.0446	3
Trigonelline	$C_7H_7NO_2$	M+H	137.0477	138.0550	138.0544	4.02	0.8	-	4
Stachydrine	$C_7H_{13}NO_2$	M+H	143.0946	144.1019	144.1015	2.81	0.6	-	4
N-Methylpelletierine	$C_9H_{17}NO$	M+H	155.1310	156.1383	156.1377	3.78	0.9	-	4
3-Hydroxystachydrine	$C_7H_{13}NO_3$	M+H	159.0895	160.0968	160.0963	3.25	0.6	-	4
Lemobiline	$C_{15}H_{17}NO_2$	M+H	243.1259	244.1332	244.1325	2.90	4.5	-	4
Arborinine	$C_{16}H_{15}NO_4$	M+H	285.1001	286.1074	286.1064	3.44	3.9	-	4
Anisodamine	$C_{17}H_{23}NO_4$	M+H	305.1627	306.1700	306.1686	4.52	4	-	4
Chelidonine	$C_{20}H_{19}NO_5$	M+H	353.1263	354.1336	354.1349	-3.67	5.5	-	4
Ancistrotectorine	$C_{26}H_{31}NO_4$	M+H	421.2253	422.2326	422.2307	4.46	6.0	-	4
Kyanmethin	$C_6H_9N_3$	M+H	123.0796	124.0869	124.0865	3.22	7.1	107.0606,810703,79.0545	3
4-Methylpelletierine	$C_{10}H_{13}NO_4$	M+H	211.0845	212.0917	212.0908	4.24	7.1	194.0811,166.0866,109.0287	3
Salsolinol	$C_9H_{11}NO_2$	M+H	165.0790	166.0863	166.0857	3.34	3.9	178.0498,132.0448,105.0338	3
Nicotinamide	$C_6H_6N_{2O}$	M+H	122.0480	123.0553	123.0548	4.00	0.7	163.0753,145.0652,117.0702	3
Kynurenate	$C_{10}H_7NO_3$	M+H	189.0426	190.0499	190.0491	4.21	4.1	162.0549,144.0438,116.0499	3
Raphanatin	$C_{16}H_{23}N_5O_6$	M+H	381.1648	382.1721	382.1704	4.47	3.8	139.0627,202.1009,145.0509	3
Deltaline	$C_{27}H_{41}NO_8$	M+H	507.2832	508.2905	508.2948	-8.47	6.6	-	4
Capnoidine	$C_{20}H_{17}NO_6$	M+H	367.1056	366.0983	366.1013	-8.17	4.8	-	4
Acetylbrowniine	$C_{27}H_{43}NO_8$	M+H	509.2989	508.2916	508.2937	-4.15	5.5	-	4

Supplemental Table S6: Tentative metabolite identification of terpenoid biofingerprints found in *D. dentatus* red and white ecotypes. SIRIUS 5.8.3 was used as an in silico fragmentation tool for metabolite annotation. Tentative identification of compounds with fragments are at level 3 using SIRIUS, otherwise at level 4 metabolite annotation.

Tentative Identification	Molecular formula	Ionization Mode	Exact mass	Theoretical mass	Experimental mass	Mass error (ppm)	t_R (min)	MS^2	Annotation Level
Perillic acid	$C_{10}H_{14}O_2$	M+H	166.0994	167.1067	167.1063	2.13	4.6	121.1011,0.93.0699,107.0859	3
(+)-Pulegone	$C_{10}H_{16}O$	M+H	152.1201	153.1274	153.1270	2.61	3.7	107.0857,97.0650,95.0860	3
Xerantholide	$C_{15}H_{18}O_3$	M+H	246.1256	247.1329	247.1322	2.83	4.7	214.1010,187.1130,149.0605	3
Alantolactone	$C_{15}H_{20}O_2$	M+H	232.1463	233.1536	233.1530	2.60	4.9	-	4
(+)-Thujopsene	$C_{15}H_{24}$	M+H	204.1878	205.1951	205.1943	3.79	4.9	-	4
Deoxyelephantopin	$C_{19}H_{20}O_6$	M+H	344.1260	345.1333	345.1319	3.95	4.7	-	4
Deoxymiroestrol	$C_{20}H_{22}O_5$	M+H	342.1467	343.1540	343.1526	4.08	5.1	-	4
(-)-Pinoresinol	$C_{20}H_{22}O_6$	M+H	358.1416	359.1489	359.1475	3.94	4.71	-	4
Niveusin C	$C_{20}H_{26}O_7$	M+H	378.1679	379.1751	379.1778	-7.04	4.09	-	4
Gibberellin A12 7-aldehyde	$C_{20}H_{28}O_3$	M+H	316.2038	317.2111	317.2098	4.10	5.7	-	4
Gibberellin A12	$C_{20}H_{28}O_4$	M+H	332.1988	333.2060	333.2047	4.01	4.8	-	4
ent-7-alpha-Hydroxykaur-16-en-19-oic acid	$C_{20}H_{30}O_3$	M+H	318.2195	319.2268	319.2256	3.67	5.9	-	4
(ent-6alpha,7alpha)-6,7-Dihydroxy-16-kauren-19-oic acid	$C_{20}H_{30}O_4$	M+H	334.2144	335.2217	335.2205	3.54	4.5	-	4
Kaurane -17,18-dioic acid	$C_{20}H_{30}O_4$	M+H	334.2144	335.2217	335.2204	3.88	4.6	153.0189, 171.0295, 139.0397	3
ent-Kaurene	$C_{20}H_{32}$	M+H	272.2504	273.2577	273.2567	3.56	6	-	4
Chromolaenide	$C_{22}H_{28}O_7$	M+H	404.1835	405.1908	405.1905	0.69	4.5	-	4
Valtratum	$C_{22}H_{30}O_8$	M+H	422.1941	423.2013	423.1974	9.32	5.4	-	4
Palmatoside G	$C_{25}H_{32}O_{10}$	M+H	492.1995	493.2068	493.2043	5.12	4	-	4
Archangelolide	$C_{29}H_{40}O_{10}$	M+H	548.2621	549.2694	549.2675	3.50	4.6	-	4

Delta7-Avenasterol	$C_{29}H_{48}O$	M+H	412.3705	413.3778	413.3759	4.58	4.9	-	4
Musaroside	$C_{30}H_{44}O_{10}$	M+H	564.2934	565.3007	565.2987	3.58	4.2	-	4
Propapyriogenin A2	$C_{30}H_{44}O_{5}$	M+H	484.3189	485.3262	485.3247	2.99	5.2	-	4
Betulonic Acid	$C_{30}H_{46}O_{3}$	M+H	454.3447	455.3520	455.3502	3.89	7	137.1332,95.0861,203.1792	3
Lupenone	$C_{30}H_{48}O$	M+H	424.3705	425.3778	425.3762	3.74	6.9	95.0860,81.0700,137.1332	3
L-Valine	$C_{5}H_{11}NO_{2}$	M+H	117.0790	118.0863	118.0859	3.01	0.6	-	4
Abscisic acid	$C_{15}H_{20}O_{4}$	M-H	264.1362	263.1289	263.1268	7.91	4.7	-	4
2-trans,6-trans-Farnesal	$C_{15}H_{24}O$	M-H	220.1827	219.1754	219.1740	6.56	7.5	-	4
Geniposidic acid	$C_{16}H_{22}O_{10}$	M-H	374.1213	373.1140	373.1115	6.70	3.7	193.0504,343.1048,181.0498	3
Secologanin	$C_{17}H_{24}O_{10}$	M-H	388.1369	387.1297	387.1282	3.80	4.14	-	4
Chaparrin	$C_{20}H_{28}O_{7}$	M-H	380.1835	379.1762	379.1755	1.92	4.9	-	4
Glaucolide B	$C_{21}H_{26}O_{10}$	M-H	438.1526	437.1453	437.1433	4.62	4.2	-	4
Buddlejoside B	$C_{22}H_{26}O_{12}$	M-H	482.1424	481.1351	481.1312	8.21	4.2	169.0138,3130573,151.0042	3
Daphnetoxin	$C_{27}H_{30}O_{8}$	M-H	482.1941	481.1868	481.1897	-6.05	4.5	-	4
Smilagenin	$C_{27}H_{44}O_{3}$	M-H	416.3290	415.3218	415.3204	3.30	6.9	-	4
Castasterone7-Oxatyphasterol	$C_{28}H_{48}O_{5}$	M-H	464.3502	463.3429	463.3408	4.53	6.5	-	4
Brassinolide	$C_{28}H_{48}O_{6}$	M-H	480.3451	479.3378	479.336	3.76	6.5	-	4
Diospyric Acid B	$C_{30}H_{46}O_{6}$	M-H	502.3294	501.3222	501.3203	3.72	5.1	439.3207,457.3303,469.3316	3
Glucosol	$C_{30}H_{48}O_{4}$	M-H	472.3553	471.3480	471.3461	4.00	5.9	427.3587,469.3331,423.3260	3

Supplemental Table S7: Tentative identification of other compounds outside of alkaloids, terpenoids and flavonoids found in both *D. dentatus* red and white ecotypes. SIRIUS was used as an in silico fragmentation tool for metabolite annotation. Tentative identification of compounds with fragments are at level 3 using SIRIUS, otherwise at level 4 metabolite annotation.

Tentative Identification	Molecular formula	Ionization Mode	Exact mass	Theoretical mass	Experimental mass	Mass error (ppm)	t_R (min)	MS^2	Annotation level
(-)-Lariciresinol	$C_{20}H_{24}O_6$	M+H	360.1573	361.1646	361.1642	1.01	4.4	-	4
Indoleacetic acid	$C_{10}H_9NO_2$	M+H	175.0633	176.0706	176.0701	2.87	5.4	-	4
Adenosine	$C_{10}H_{13}N_5O_4$	M+H	267.0968	268.1040	268.1029	4.22	1.7	-	4
1-O-Sinapoyl-beta-D-glucose	$C_{17}H_{22}O_{10}$	M+H	386.1213	387.1286	387.1273	3.29	4.03	-	4
Nordihydroguaiaretic acid	$C_{18}H_{22}O_4$	M+H	302.1518	303.1591	303.1579	3.91	5.7	-	4
Podophyllotoxone	$C_{22}H_{20}O_8$	M+H	412.1158	413.1231	413.1214	4.10	4.43	-	4
Lutein	$C_{40}H_{56}O_2$	M+H	568.4280	569.4353	569.4364	-1.92	4.3	-	4
Anthragallol	$C_{14}H_8O_5$	M+H	256.0372	257.0444	257.0435	3.70	4.5	-	4
Biflorin	$C_{16}H_{18}O_9$	M+H	354.0951	355.1024	355.1011	3.54	4.3	-	4
Gartanin	$C_{23}H_{24}O_6$	M+H	396.1573	397.1646	397.163	3.94	5.6	-	4
Marchantin A	$C_{28}H_{24}O_5$	M+H	440.1624	441.1697	441.1717	-4.65	4.3	-	4
Scorzocreticin	$C_{16}H_{14}O_5$	M+H	286.0841	287.0914	287.0905	3.13	4.7	269.0815,257.0826,227.0717	3
Caffeate	$C_9H_8O_4$	M-H	180.0423	179.0350	179.0343	3.81	4.3	-	4
1-O-Sinapoyl-beta-D-glucose	$C_{17}H_{22}O_{10}$	M-H	386.1213	385.1140	385.1126	3.69	4.4	-	4
5-Hydroxyconiferaldehyde	$C_{10}H_{10}O_5$	M-H	210.0528	209.0455	209.0447	4.05	4.4	-	4
Chlorogenate	$C_{16}H_{18}O_9$	M-H	354.0951	353.0878	353.0858	5.68	4.4	-	4
3-Hydroxypropanoate	$C_3H_6O_3$	M-H	90.0317	89.0244	89.0241	3.56	0.6	-	4
2-Oxobutanoate	$C_4H_6O_3$	M-H	102.0317	101.0244	101.0239	5.12	0.6	-	4
Succinate	$C_4H_6O_4$	M-H	118.0266	117.0193	117.0189	3.69	0.8	-	4
cis-Aconitate	$C_6H_6O_6$	M-H	174.0164	173.0092	173.0083	5.0	0.6	-	4

Isocitrate	$C_6H_8O_7$	M-H	192.0270	191.0197	191.019	3.80	0.6	-	4
Chorismate	$C_{10}H_{10}O_6$	M-H	226.0477	225.0405	225.0397	3.38	4	-	4
L-Arogenic acid	$C_{10}H_{13}NO_5$	M-H	163.0997	162.0924	162.0922	1.46	3.9	-	4
Oxalic acid	$C_2H_2O_4$	M-H	89.9953	88.9880	88.9877	3.73	1	-	4
Gallate	$C_7H_6O_5$	M-H	170.0215	169.0142	169.0134	5.01	0.5	125.0245,97.0290,79.0186	3
2-hydroxy-5-[(3,4,5-trihydroxyoxan-2-yl) oxy]benzoic acid	$C_{12}H_{14}O_8$	M-H	286.0689	285.0616	285.0599	5.96	3.9	152.0106,108.0213,109.0291	3
Lyoniside	$C_{27}H_{36}O_{12}$	M-H	552.2207	551.2134	551.2095	7.07	4.3	373.1282,404.1492,59.0137	3
Gentisic acid	$C_7H_6O_4$	M-H	154.0266	153.0193	153.0178	6.74	4.1	109.0292,110.0010,137.9956	3
Atraric Acid	$C_{10}H_{12}O_4$	M-H	196.0736	195.0663	195.0648	7.59	5.3	151.0765,167.0350,136.0528	3

Supplemental Table S8: Pathway impacts of biochemical pathways in *Doliocarpus dentatus* white ecotype as a result of using the mummichog algorithm used in Pathway analysis module in MetaboAnalyst 5.0

Pathways	Total	Expected	Hits	Raw p	-Log10(p)	Holm adjust	False Discovery Rate	Impact
Flavonoid biosynthesis	47	8.2717	23	5.22E-07	6.2822	5.01E-05	5.01E-05	0.35771
Steroid biosynthesis	45	7.9198	18	0.000271	3.5674	0.025183	0.0064988	0.45432
Phenylpropanoid biosynthesis	46	8.0958	14	0.021621	1.6651	1	0.13837	0.49931
Valine, leucine and isoleucine biosynthesis	22	3.8719	13	1.30E-05	4.8856	0.0012362	0.0006246	0.43383
Ubiquinone and other terpenoid-quinone biosynthesis	38	6.6878	13	0.009532	2.0208	0.81971	0.083184	0.44435
Galactose metabolism	27	4.7519	11	0.003727	2.4287	0.32795	0.039752	0.48491
alpha-Linolenic acid metabolism	28	4.9278	11	0.005197	2.2842	0.45218	0.049895	0.65224
Amino sugar and nucleotide sugar metabolism	50	8.7997	11	0.25317	0.59658	1	0.90017	0.03673
Diterpenoid biosynthesis	28	4.9278	10	0.01618	1.791	1	0.11949	0.38809
Tyrosine metabolism	16	2.8159	9	0.000515	3.2878	0.047421	0.0098967	0.47297
Cutin, suberine and wax biosynthesis	18	3.1679	9	0.001575	2.8029	0.1417	0.021593	0.4375
Fructose and mannose metabolism	20	3.5199	8	0.014897	1.8269	1	0.11918	0.12586
Sesquiterpenoid and triterpenoid biosynthesis	24	4.2239	8	0.045824	1.3389	1	0.27494	0.36175
Biosynthesis of unsaturated fatty acids	22	3.8719	7	0.07579	1.1204	1	0.40421	0
Pantothenate and CoA biosynthesis	23	4.0479	7	0.093272	1.0302	1	0.4477	0.2203
Brassinosteroid biosynthesis	26	4.5759	7	0.1577	0.80217	1	0.6308	0.15239
Fatty acid biosynthesis	56	9.8557	7	0.88905	0.051076	1	1	0.01123
Biosynthesis of secondary metabolites - other antibiotics	6	1.056	6	2.83E-05	4.5477	0.0026631	0.00090658	1
Flavone and flavonol biosynthesis	10	1.7599	6	0.003146	2.5023	0.27995	0.037745	0.85
Ascorbate and aldarate metabolism	18	3.1679	6	0.080095	1.0964	1	0.40469	0.26865
Citrate cycle (TCA cycle)	20	3.5199	6	0.12342	0.9086	1	0.53858	0.18262
Glyoxylate and dicarboxylate metabolism	29	5.1038	6	0.40343	0.39424	1	1	0.30723
Glycine, serine and threonine metabolism	33	5.8078	6	0.53731	0.26977	1	1	0.10432

Carotenoid biosynthesis	43	7.5678	6	0.79613	0.099017	1	1	0.0365	
Purine metabolism	63	11.088	6	0.97766	0.0098126	1	1	0.1955	
C5-Branched dibasic acid metabolism	6	1.056	5	0.00084	3.0759	0.076416	0.013436	1	
Butanoate metabolism	17	2.9919	5	0.16454	0.78373	1	0.63183	0	
Phenylalanine, tyrosine and tryptophan biosynthesis	22	3.8719	5	0.34249	0.46535	1	1	0.21672	
Starch and sucrose metabolism	22	3.8719	5	0.34249	0.46535	1	1	0.51292	
Aminoacyl-tRNA biosynthesis	46	8.0958	5	0.92905	0.031959	1	1	0.05556	
Glucosinolate biosynthesis	65	11.44	5	0.99418	0.002536	1	1	0	
Phenylalanine metabolism	11	1.9359	4	0.1114	0.9531	1	0.50927	0.23529	
Alanine, aspartate and glutamate metabolism	22	3.8719	4	0.55939	0.25228	1	1	0.07554	
Pyruvate metabolism	22	3.8719	4	0.55939	0.25228	1	1	0.24039	
Tryptophan metabolism	28	4.9278	4	0.75405	0.1226	1	1	0.2037	
Cyanoamino acid metabolism	29	5.1038	4	0.77905	0.10844	1	1	0.23729	
Terpenoid backbone biosynthesis	30	5.2798	4	0.802	0.095826	1	1	0.14525	
Valine, leucine and isoleucine degradation	37	6.5118	4	0.9136	0.039246	1	1	0.00991	
Cysteine and methionine metabolism	46	8.0958	4	0.97377	0.011545	1	1	0.08428	
Linoleic acid metabolism	4	0.70398	3	0.018775	1.7264	1	0.12874	1	
Isoquinoline alkaloid biosynthesis	6	1.056	3	0.071241	1.1473	1	0.4023	0	
Stilbenoid, diarylheptanoid and gingerol biosynthesis	8	1.408	3	0.15231	0.81729	1	0.6308	0.39705	
Arachidonic acid metabolism	12	2.1119	3	0.35578	0.44881	1	1	0	
Pentose and glucuronate interconversions	16	2.8159	3	0.55408	0.25643	1	1	0.15625	
Arginine biosynthesis	18	3.1679	3	0.63873	0.19468	1	1	0.12816	
beta-Alanine metabolism	18	3.1679	3	0.63873	0.19468	1	1	0.31746	
Glycerolipid metabolism	21	3.6959	3	0.74343	0.12876	1	1	0.00426	
Zeatin biosynthesis	21	3.6959	3	0.74343	0.12876	1	1	0.01491	
Thiamine metabolism	22	3.8719	3	0.77249	0.11211	1	1	0.20231	
Glycolysis / Gluconeogenesis	26	4.5759	3	0.86292	0.06403	1	1	0.1215	
Inositol phosphate metabolism	28	4.9278	3	0.89502	0.048167	1	1	0.10251	
Limonene and pinene degradation	5	0.87997	2	0.21423	0.66911	1	0.79102	0	
Monobactam biosynthesis	8	1.408	2	0.42454	0.37208	1	1	0	
One carbon pool by folate	8	1.408	2	0.42454	0.37208	1	1	0	
Monoterpenoid biosynthesis	9	1.584	2	0.48866	0.31099	1	1	0	

Caffeine metabolism	10	1.7599	2	0.54813	0.26112	1	1	0	
Selenocompound metabolism	13	2.2879	2	0.69631	0.1572	1	1	0.2437	
Sphingolipid metabolism	17	2.9919	2	0.82924	0.081322	1	1	0.24038	
Pentose phosphate pathway	19	3.3439	2	0.87381	0.058585	1	1	0	
Carbon fixation in photosynthetic organisms	21	3.6959	2	0.90746	0.042172	1	1	0.03607	
Glycerophospholipid metabolism	37	6.5118	2	0.99357	0.0028005	1	1	0.06318	
Betalain biosynthesis	3	0.52798	1	0.44075	0.3558	1	1	1	
Tropane, piperidine and pyridine alkaloid biosynthesis	8	1.408	1	0.78832	0.1033	1	1	0	
Lysine biosynthesis	9	1.584	1	0.82578	0.083138	1	1	0.2027	
Vitamin B6 metabolism	11	1.9359	1	0.88203	0.054517	1	1	0.03205	
Riboflavin metabolism	11	1.9359	1	0.88203	0.054517	1	1	0.06667	
Nicotinate and nicotinamide metabolism	13	2.2879	1	0.92017	0.036133	1	1	0	
Lysine degradation	18	3.1679	1	0.97	0.013227	1	1	0	
Propanoate metabolism	20	3.5199	1	0.97974	0.0088884	1	1	0.08554	
Fatty acid elongation	23	4.0479	1	0.98877	0.0049048	1	1	0	
Glutathione metabolism	26	4.5759	1	0.99378	0.0027085	1	1	0	
Phosphatidylinositol signaling system	26	4.5759	1	0.99378	0.0027085	1	1	0.03285	
Folate biosynthesis	27	4.7519	1	0.9949	0.0022221	1	1	0.02624	
Arginine and proline metabolism	34	5.9838	1	0.99872	0.00055462	1	1	0.0122	
Fatty acid degradation	37	6.5118	1	0.9993	0.00030544	1	1	0	
Pyrimidine metabolism	38	6.6878	1	0.99942	0.00025029	1	1	0	
Porphyrin and chlorophyll metabolism	48	8.4477	1	0.99992	3.39E-05	1	1	0.07537	

Supplemental Table S9: Biochemical pathway impacts observed in *Doliocarpus dentatus* red ecotype as a result of using the mummichog algorithm used in Pathway analysis module in MetaboAnalyst 5.0

Pathways	Total	Expected	Hits	Raw p		Holm adjust	False Discovery Rate	Impact	
Sesquiterpenoid and triterpenoid biosynthesis	24	5.648	19	1.00E-08	7.9991	9.62E-07	9.62E-07	0.57797	
Galactose metabolism	27	6.354	20	2.91E-08	7.5357	2.77E-06	1.40E-06	0.82797	
Flavonoid biosynthesis	47	11.061	25	7.37E-06	5.1325	0.00069283	0.000236	0.4785	
Ascorbate and aldarate metabolism	18	4.236	12	0.00011168	3.952	0.010386	0.00268	0.64179	
Biosynthesis of secondary metabolites - other antibiotics	6	1.412	6	0.00016434	3.7843	0.015119	0.003155	1	
Steroid biosynthesis	45	10.59	21	0.0004642	3.3333	0.042243	0.006488	0.62018	
Fructose and mannose metabolism	20	4.7067	12	0.00047307	3.3251	0.042576	0.006488	0.65819	
Flavone and flavonol biosynthesis	10	2.3533	7	0.002327	2.6332	0.2071	0.027924	0.65	
Ubiquinone and other terpenoid-quinone biosynthesis	38	8.9427	17	0.0028877	2.5395	0.25411	0.030692	0.55568	
Cutin, suberine and wax biosynthesis	18	4.236	10	0.0031971	2.4952	0.27815	0.030692	0.75	
Valine, leucine and isoleucine biosynthesis	22	5.1773	11	0.0058125	2.2356	0.49987	0.0465	0.53039	
Starch and sucrose metabolism	22	5.1773	11	0.0058125	2.2356	0.49987	0.0465	0.74456	
alpha-Linolenic acid metabolism	28	6.5893	12	0.017648	1.7533	1	0.13032	0.60278	
Riboflavin metabolism	11	2.5887	6	0.02533	1.5964	1	0.17369	0.75557	
Citrate cycle (TCA cycle)	20	4.7067	9	0.027489	1.5608	1	0.17593	0.39412	
C5-Branched dibasic acid metabolism	6	1.412	4	0.030102	1.5214	1	0.18061	0.5	
Linoleic acid metabolism	4	0.94134	3	0.04271	1.3695	1	0.23746	1	
Diterpenoid biosynthesis	28	6.5893	11	0.044525	1.3514	1	0.23746	0.38321	
Amino sugar and nucleotide sugar metabolism	50	11.767	17	0.058165	1.2353	1	0.28261	0.43695	
Tyrosine metabolism	16	3.7653	7	0.058878	1.2301	1	0.28261	0.27027	
Butanoate metabolism	17	4.0007	7	0.080576	1.0938	1	0.36835	0.22727	
Glyoxylate and dicarboxylate metabolism	29	6.8247	10	0.12049	0.91905	1	0.50883	0.40321	
Alanine, aspartate and glutamate metabolism	22	5.1773	8	0.12191	0.91397	1	0.50883	0.46044	
Arachidonic acid metabolism	12	2.824	5	0.12798	0.89284	1	0.51194	0	
Pentose phosphate pathway	19	4.4713	7	0.13585	0.86694	1	0.52167	0.36819	
Pantothenate and CoA biosynthesis	23	5.4127	8	0.15051	0.82244	1	0.55572	0.24838	
Caffeine metabolism	10	2.3533	4	0.19027	0.72064	1	0.6765	0	
Biosynthesis of unsaturated fatty acids	22	5.1773	7	0.24409	0.61245	1	0.83547	0	
Glycolysis / Gluconeogenesis	26	6.1187	8	0.25238	0.59794	1	0.83547	0.24829	

Phenylpropanoid biosynthesis	46	10.825	13	0.27097	0.56708	1	0.8671	0.44832	
Pentose and glucuronate interconversions	16	3.7653	5	0.31708	0.49883	1	0.98193	0.25	
Thiamine metabolism	22	5.1773	6	0.41848	0.37833	1	1	0.47399	
Phenylalanine metabolism	11	2.5887	3	0.49894	0.30195	1	1	0.23529	
Inositol phosphate metabolism	28	6.5893	7	0.50041	0.30068	1	1	0.14853	
Phosphonate and phosphinate metabolism	7	1.6473	2	0.51818	0.28552	1	1	0	
Valine, leucine and isoleucine degradation	37	8.7073	9	0.51908	0.28476	1	1	0.05889	
Propanoate metabolism	20	4.7067	5	0.52492	0.27991	1	1	0.22404	
Cyanoamino acid metabolism	29	6.8247	7	0.54196	0.26604	1	1	0.20339	
Zeatin biosynthesis	21	4.942	5	0.57305	0.24181	1	1	0.02847	
Carbon fixation in photosynthetic organisms	21	4.942	5	0.57305	0.24181	1	1	0.16543	
Sphingolipid metabolism	17	4.0007	4	0.5942	0.22607	1	1	0.40865	
Monobactam biosynthesis	8	1.8827	2	0.5959	0.22483	1	1	0	
One carbon pool by folate	8	1.8827	2	0.5959	0.22483	1	1	0	
Brassinosteroid biosynthesis	26	6.1187	6	0.59863	0.22284	1	1	0.11429	
Pyruvate metabolism	22	5.1773	5	0.61851	0.20865	1	1	0.40463	
beta-Alanine metabolism	18	4.236	4	0.64322	0.19164	1	1	0.38889	
Synthesis and degradation of ketone bodies	4	0.94134	1	0.65854	0.18142	1	1	0	
Indole alkaloid biosynthesis	4	0.94134	1	0.65854	0.18142	1	1	0.5	
Monoterpenoid biosynthesis	9	2.118	2	0.66382	0.17795	1	1	0	
Lysine biosynthesis	9	2.118	2	0.66382	0.17795	1	1	0.29729	
Glycine, serine and threonine metabolism	33	7.766	7	0.69076	0.16067	1	1	0.10432	
Limonene and pinene degradation	5	1.1767	1	0.73911	0.13129	1	1	0	
Glycerolipid metabolism	21	4.942	4	0.7657	0.11594	1	1	0.00426	
Anthocyanin biosynthesis	11	2.5887	2	0.77187	0.11246	1	1	0	
Vitamin B6 metabolism	11	2.5887	2	0.77187	0.11246	1	1	0.03205	
Phenylalanine, tyrosine and tryptophan biosynthesis	22	5.1773	4	0.79841	0.097775	1	1	0.28178	
Isoquinoline alkaloid biosynthesis	6	1.412	1	0.80071	0.096522	1	1	0	
Arginine biosynthesis	18	4.236	3	0.83362	0.079031	1	1	0.2136	
Nicotinate and nicotinamide metabolism	13	3.0593	2	0.84836	0.071418	1	1	0.30707	
Tropane, piperidine and pyridine alkaloid biosynthesis	8	1.8827	1	0.88379	0.053651	1	1	0	
Stilbenoid, diarylheptanoid and gingerol biosynthesis	8	1.8827	1	0.88379	0.053651	1	1	0.13235	
Phosphatidylinositol signaling system	26	6.1187	4	0.89415	0.048589	1	1	0.03545	

Sulfur metabolism	15	3.53	2	0.90082	0.045364	1	1	0.10774	
Carotenoid biosynthesis	43	10.119	7	0.91147	0.040259	1	1	0.07053	
Tryptophan metabolism	28	6.5893	4	0.92499	0.033863	1	1	0.1713	
Terpenoid backbone biosynthesis	30	7.06	4	0.94751	0.023414	1	1	0.17239	
Nitrogen metabolism	12	2.824	1	0.96058	0.017465	1	1	0	
Selenocompound metabolism	13	3.0593	1	0.96994	0.013257	1	1	0	
Fatty acid biosynthesis	56	13.179	8	0.97158	0.01252	1	1	0.01123	
Aminoacyl-tRNA biosynthesis	46	10.825	6	0.97634	0.010401	1	1	0.05556	
Cysteine and methionine metabolism	46	10.825	6	0.97634	0.010401	1	1	0.17084	
Purine metabolism	63	14.826	9	0.97762	0.009828	1	1	0.11117	
Histidine metabolism	15	3.53	1	0.98252	0.0076586	1	1	0.13953	
Glutathione metabolism	26	6.1187	2	0.99199	0.0034919	1	1	0.05046	
Lysine degradation	18	4.236	1	0.99226	0.0033732	1	1	0	
Arginine and proline metabolism	34	8.0013	3	0.99341	0.0028701	1	1	0.09001	
Glucosinolate biosynthesis	65	15.297	8	0.99344	0.002859	1	1	0.00154	
Folate biosynthesis	27	6.354	2	0.9937	0.0027463	1	1	0	
Pyrimidine metabolism	38	8.9427	3	0.9973	0.0011744	1	1	0.13096	
Fatty acid elongation	23	5.4127	1	0.99802	0.00086099	1	1	0	
N-Glycan biosynthesis	35	8.2367	2	0.99911	0.00038711	1	1	0.07466	
Porphyrin and chlorophyll metabolism	48	11.296	3	0.99973	0.00011525	1	1	0.09842	
Fatty acid degradation	37	8.7073	1	0.99996	1.84E-05	1	1	0	
Glycerophospholipid metabolism	37	8.7073	1	0.99996	1.84E-05	1	1	0.02629	

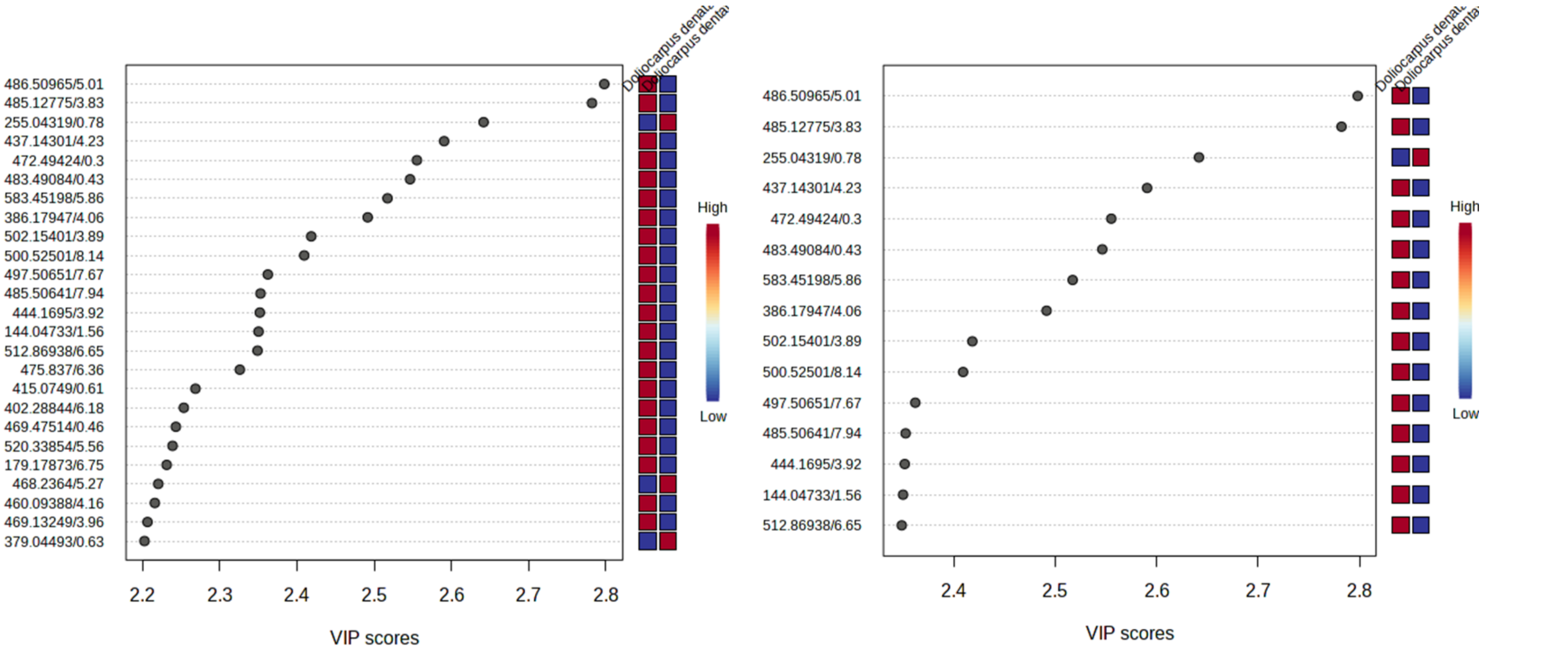

Supplemental Figure S1: (B) VIP scores for PLS-DA showing features responsible for divergence from PCA analysis. (A) represents data in positive ionization mode and (B) negative ionization mode.

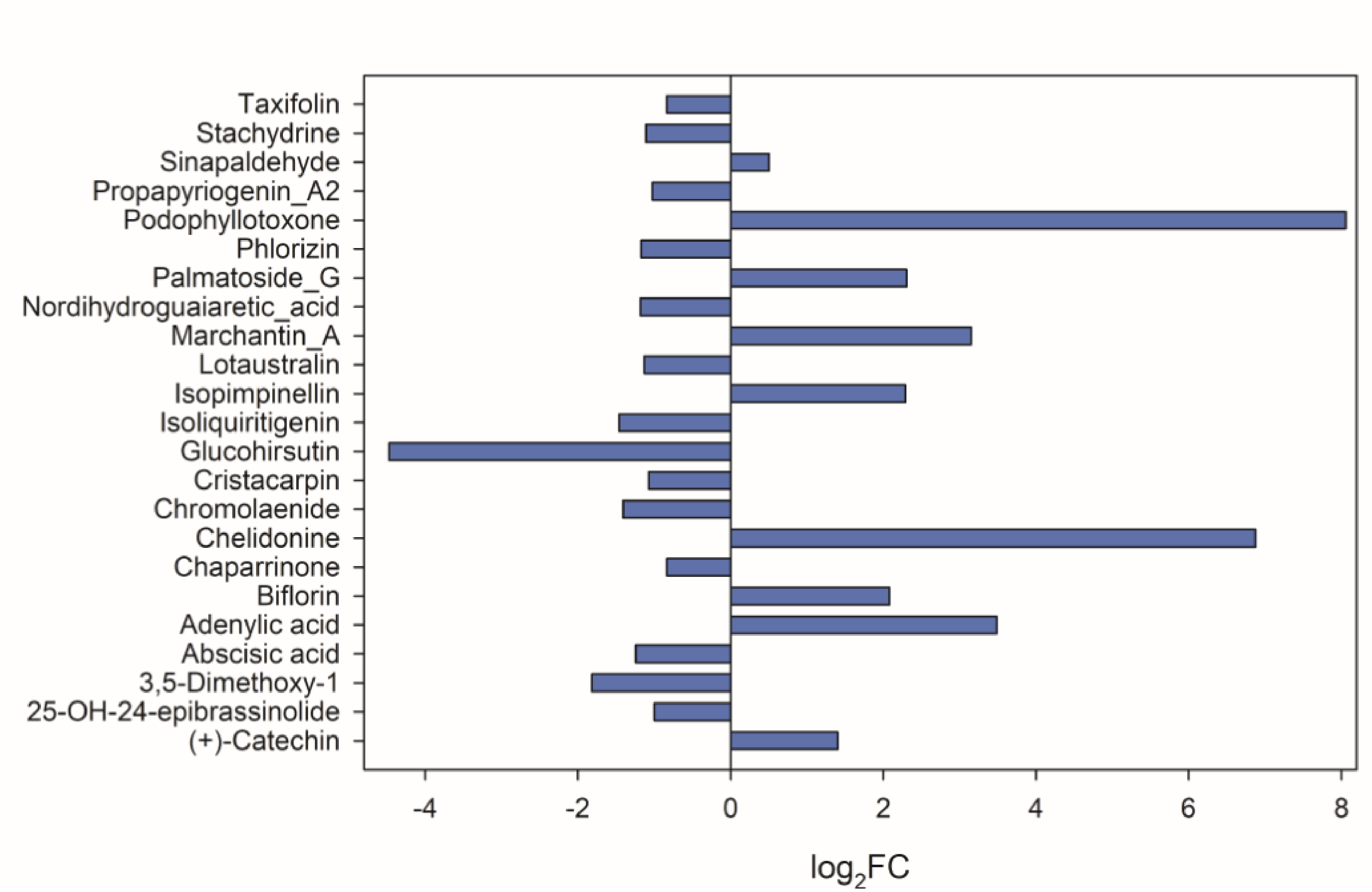

Supplemental Figure S2: Bar chart showing up-or-downregulated select putative compounds at level 4 metabolite annotation. Metabolite annotation was done via MetaboAnalyst 5.0, MetaboQuest and linked to KEGG. Xcalibur Software (v.4.1) was used to confirm masses and peak quality in sample replicates.

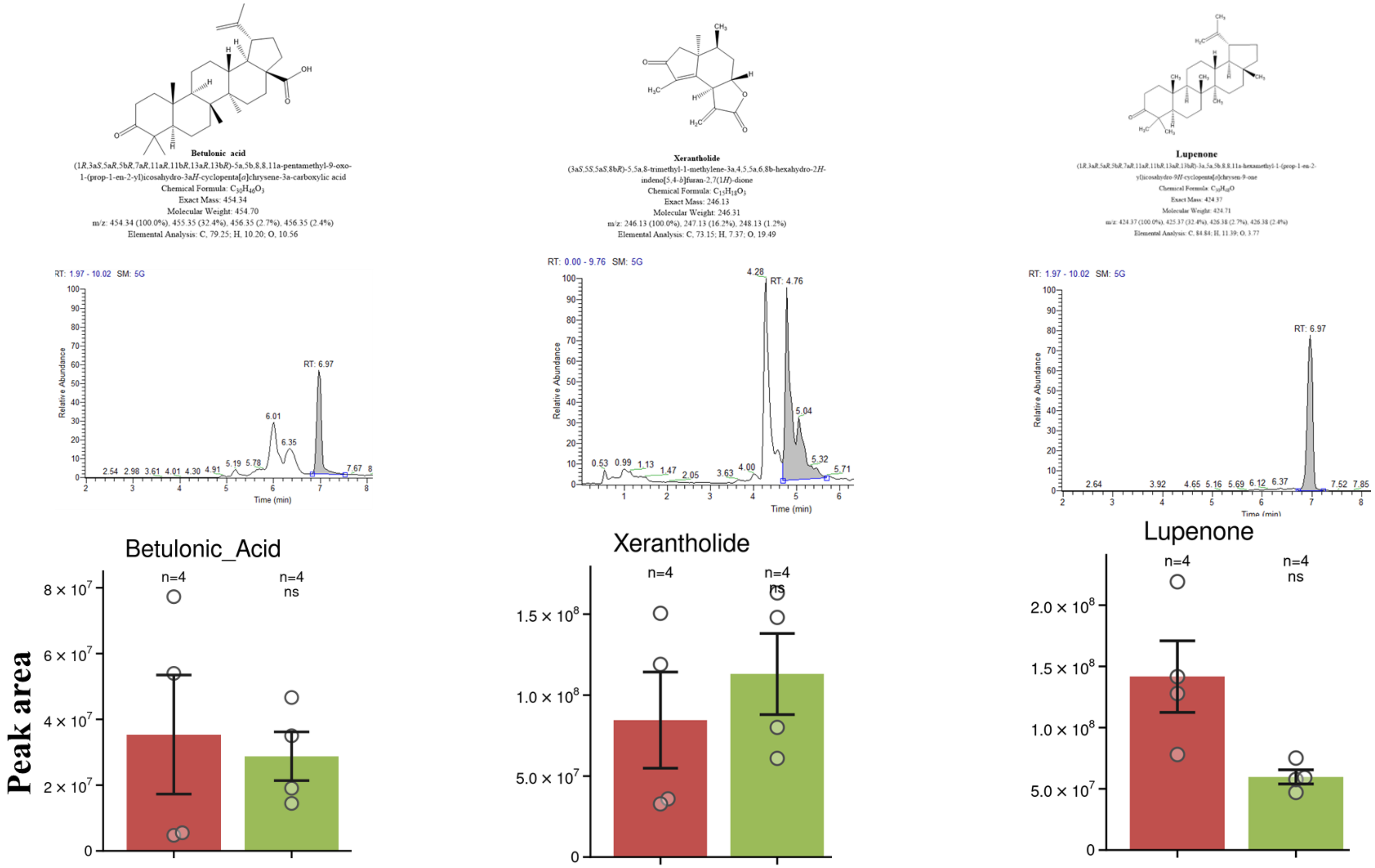

Supplemental Figure S3: Select terpenoid biomarkers showing molecular structure and formulae, chromatogram peak region found, and integrated peak areas. For integrated peak areas, red and green denote red and white Capadulla respectively.

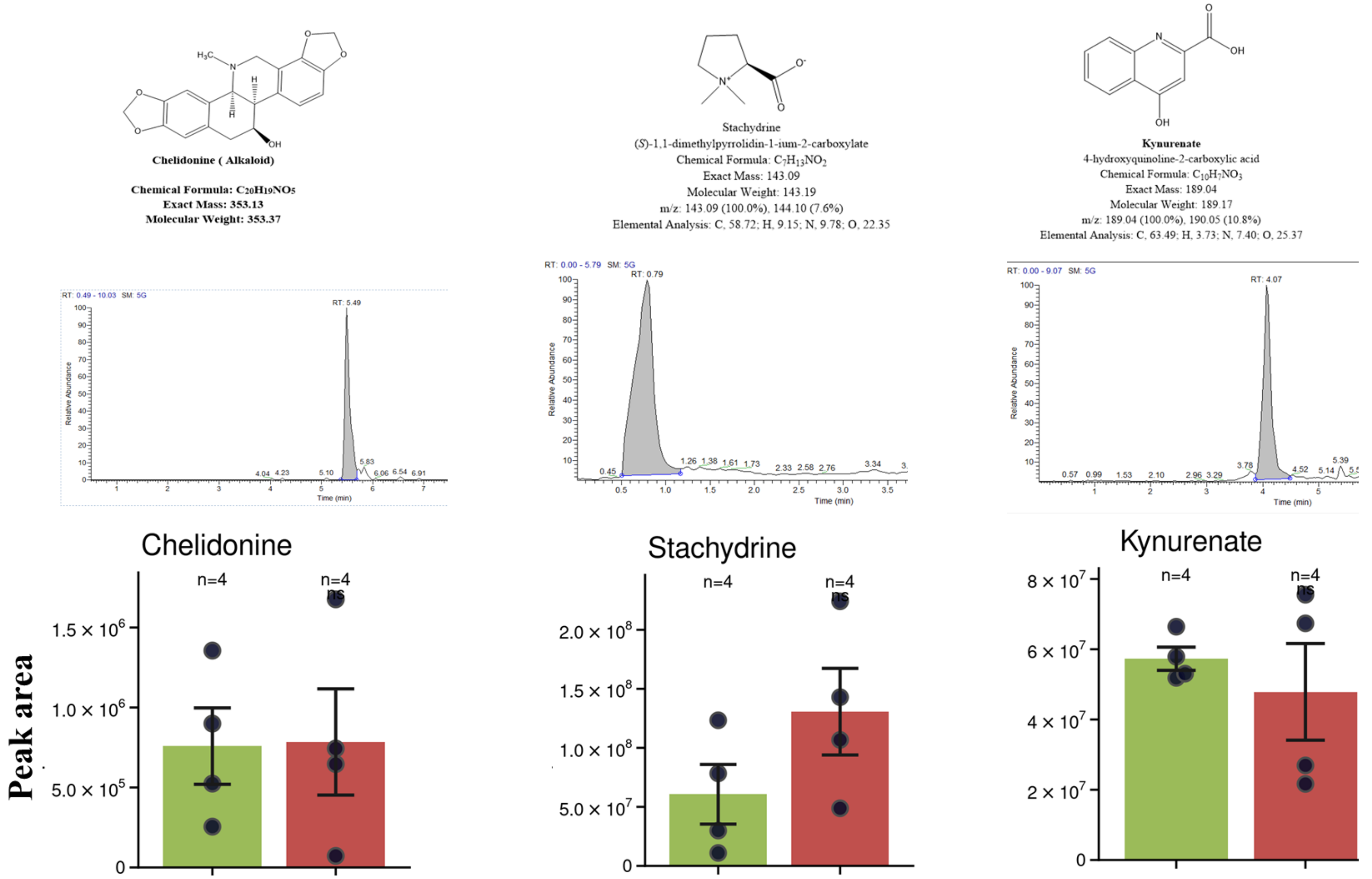

Supplemental Figure S4: Select alkaloid biomarkers showing molecular structure and formulae, chromatogram peak region found, and integrated peak areas. For integrated peak areas, red and green denote red and white Capadulla respectively.

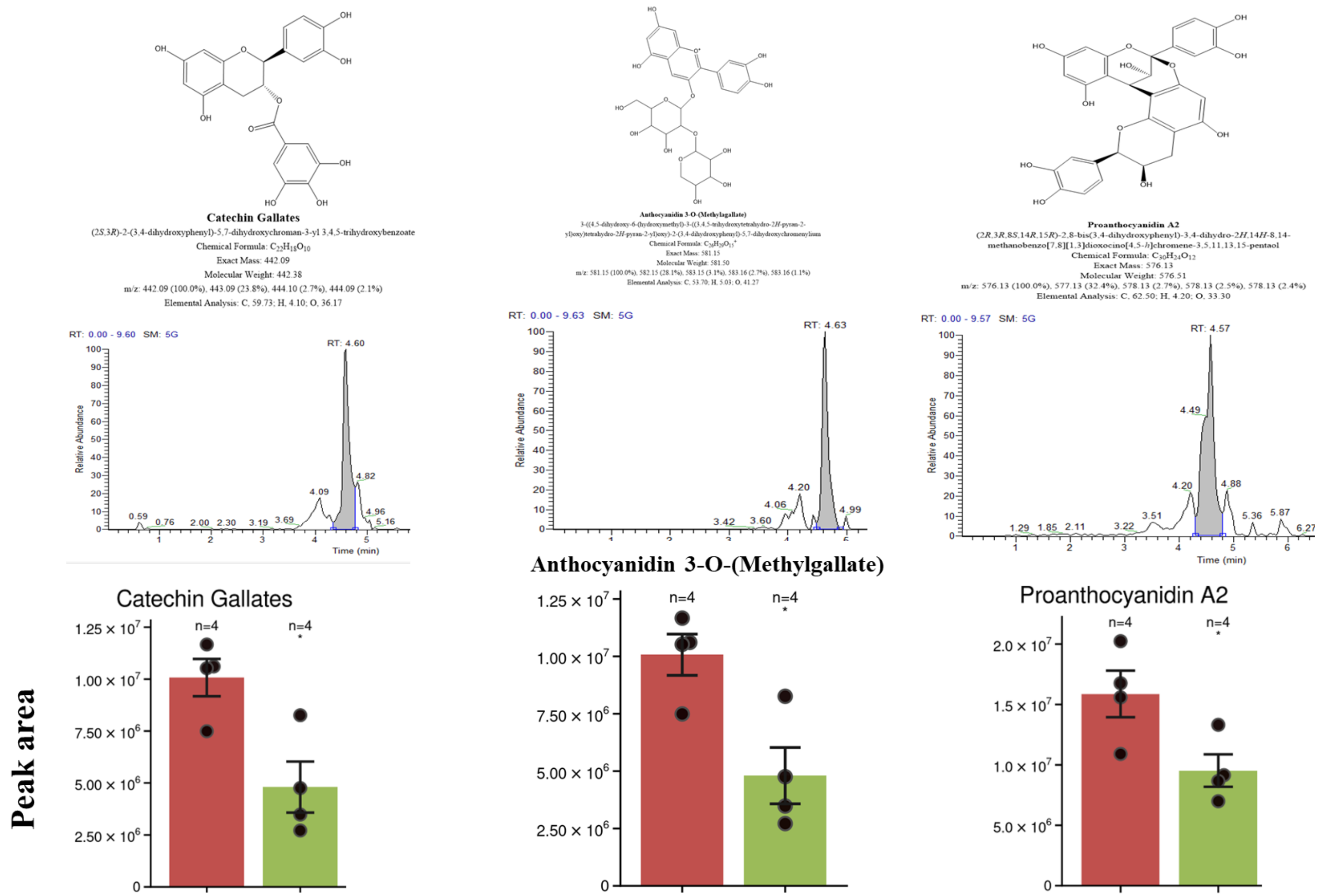

Supplemental Figure S5: Select phenolic biomarkers showing molecular structure and formulae, chromatogram peak region found, and integrated peak areas. For integrated peak areas, red and green denote red and white Capadulla respectively.

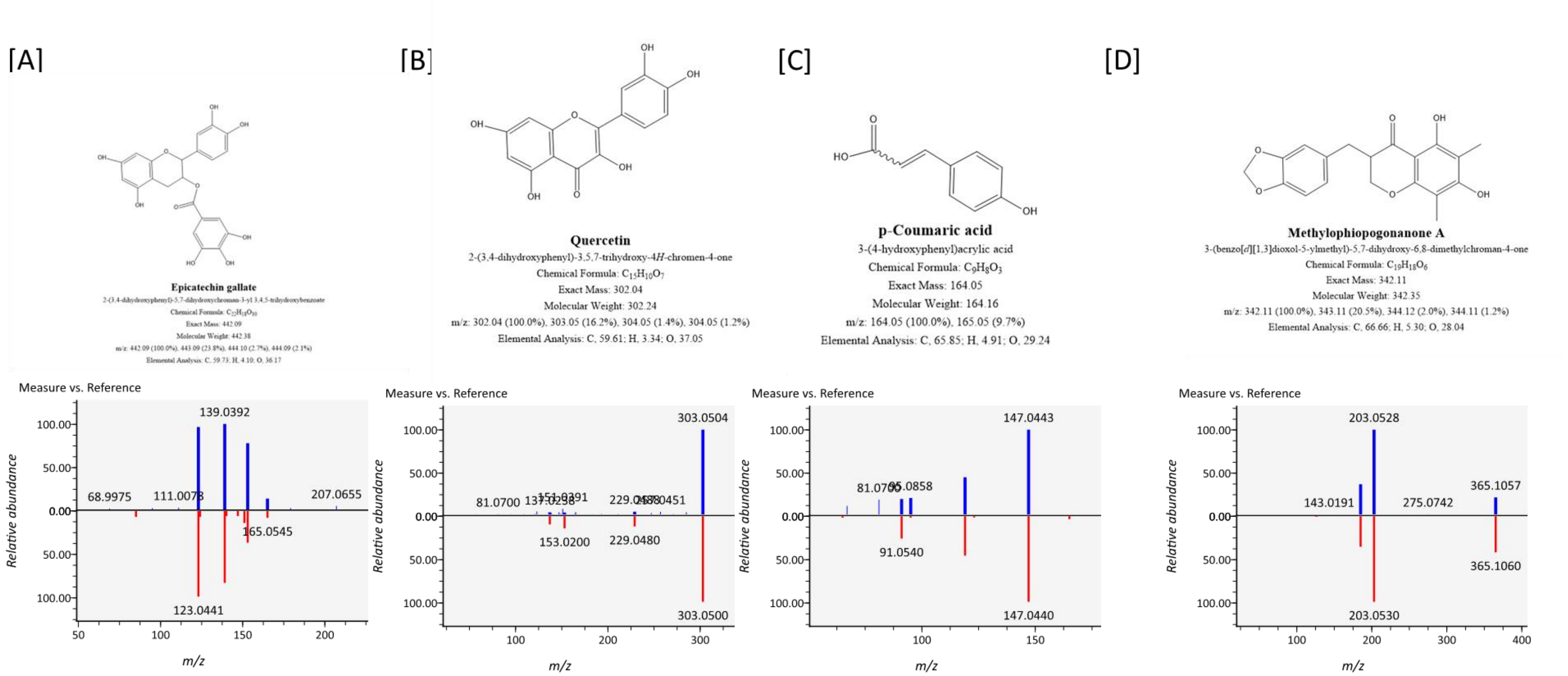

Supplemental Figure S6A: Select level 2 tentative identification of flavonoid compounds in positive ionization mode using MS-DIAL 5.1.

[E]

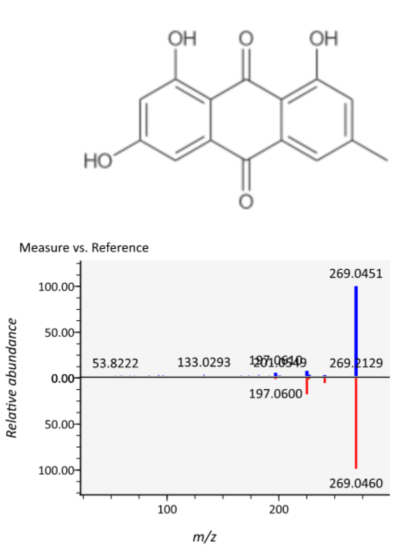

Emodin

[F]

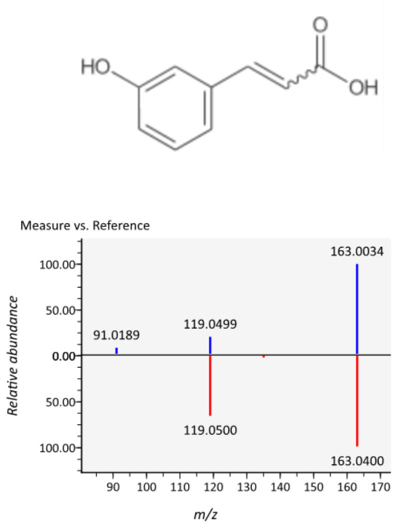

3-Coumaric acid

[G]

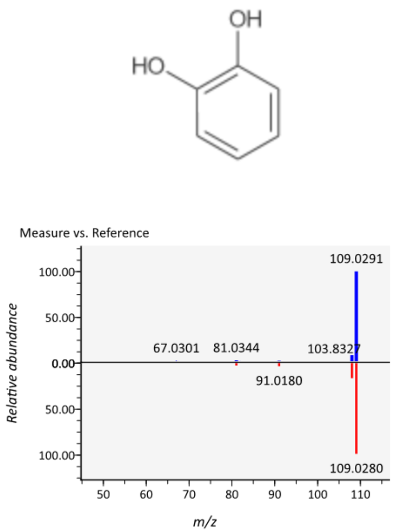

Catechol

[H]

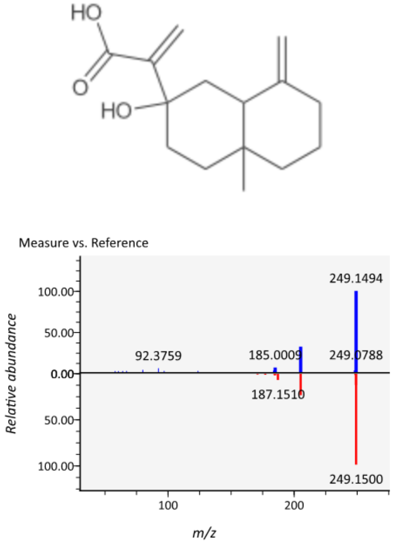

2-[(2S,4aR,8aS)-2-hydroxy-4a-methyl-8-methylidene-3,4,5,6,7,8a-hexahydro-1H-naphthalen-2-yl]prop-2-enoic acid

Supplemental Figure S6B: Select level 2 tentative identification of flavonoid compounds in positive ionization mode using MS-DIAL 5.1.

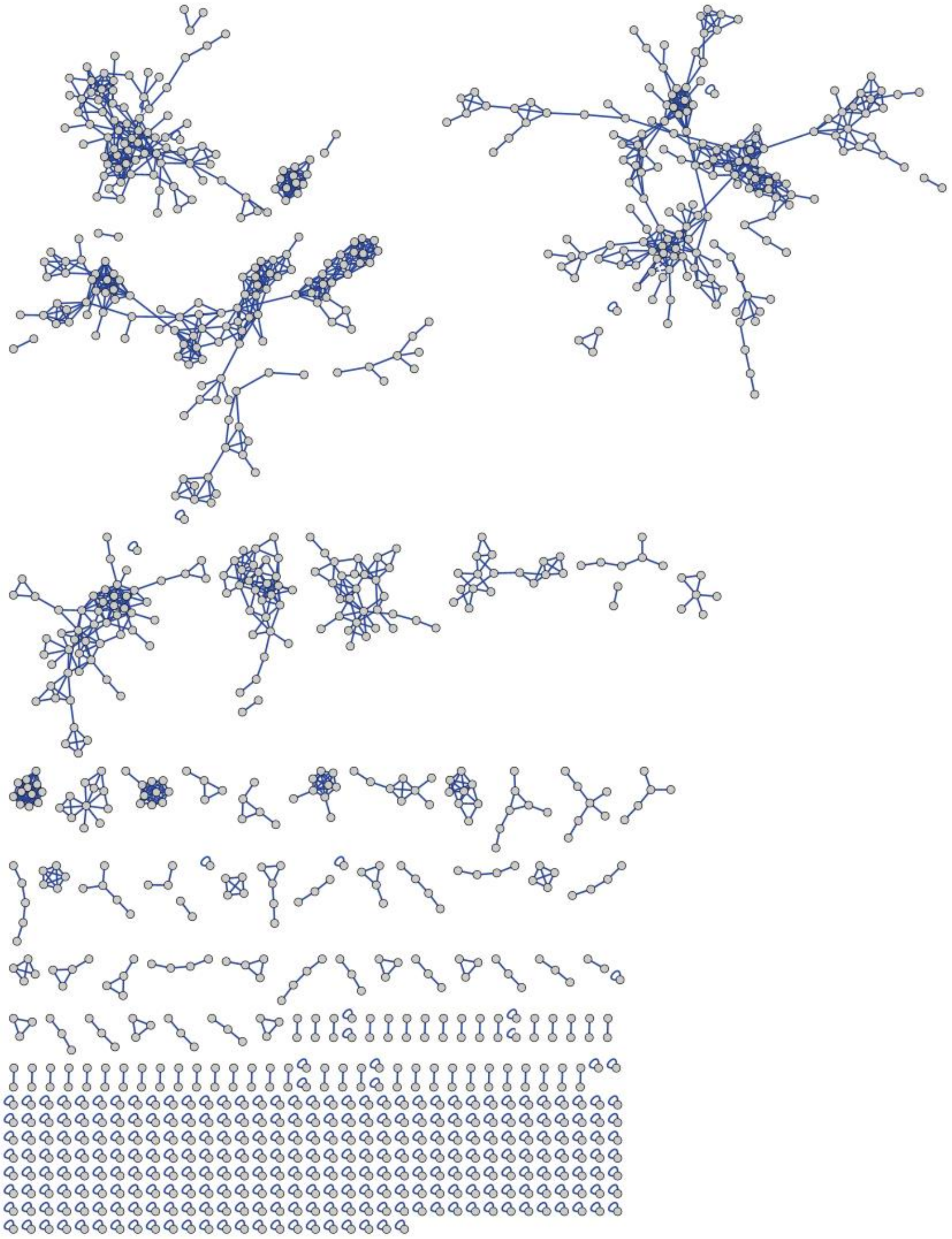

Supplemental Figure S7: Merged polarity network analysis of classical molecular networking for *D. dentatus* ecotypes using positive and negative ionization modes.

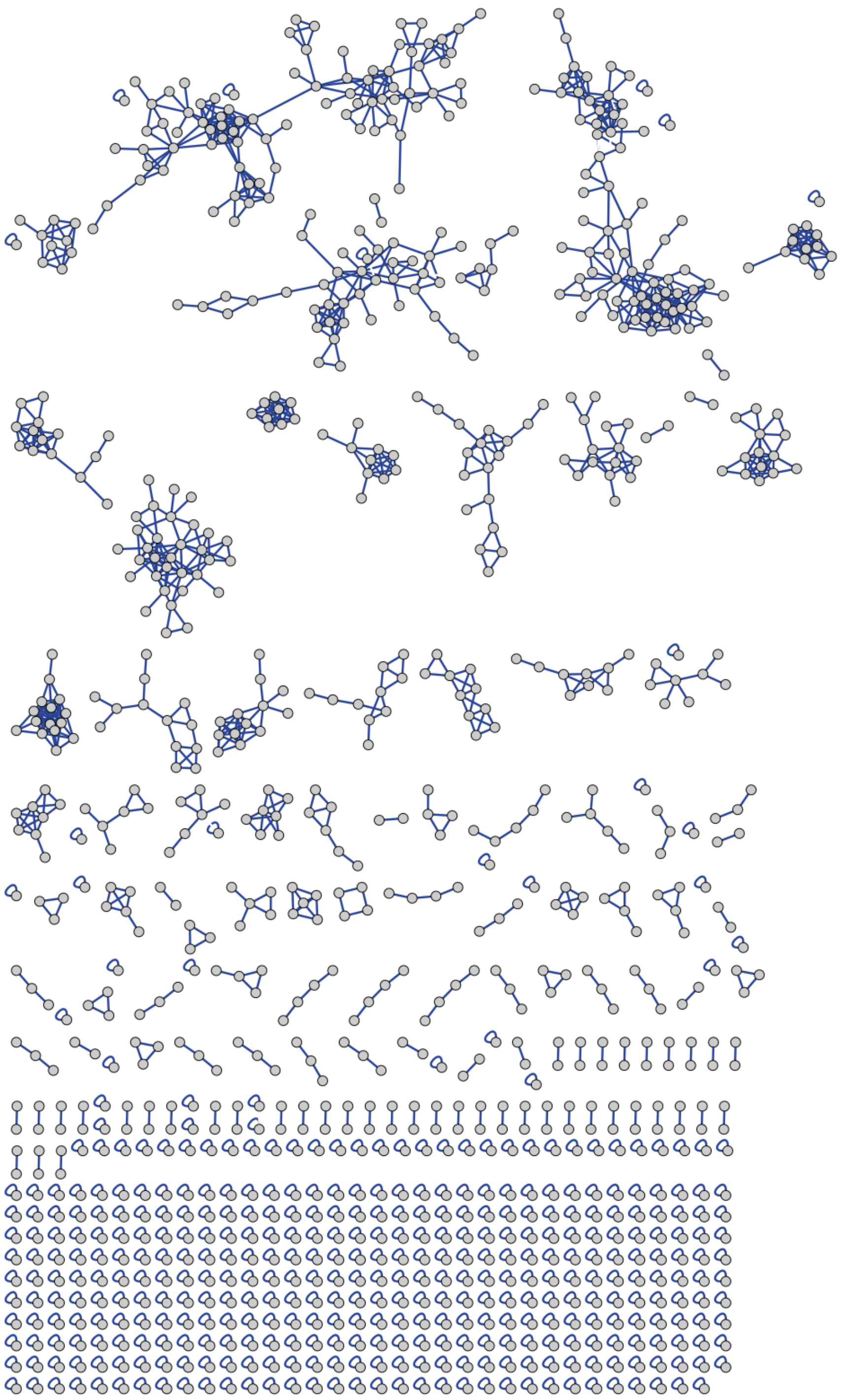

Supplemental Figure S8: Merged polarity network analysis of feature-based molecular networking for *D. dentatus* ecotypes using positive and negative ionization modes.
